# Supplementary figures and images for: Transcriptome-wide gene-gene interaction associations elucidate pathways and functional enrichment of complex traits
Source: PLoS Genet. 2023 May 22;19(5):e1010693. doi: 10.1371/journal.pgen.1010693 (PMC10237671; doi:10.1371/journal.pgen.1010693)

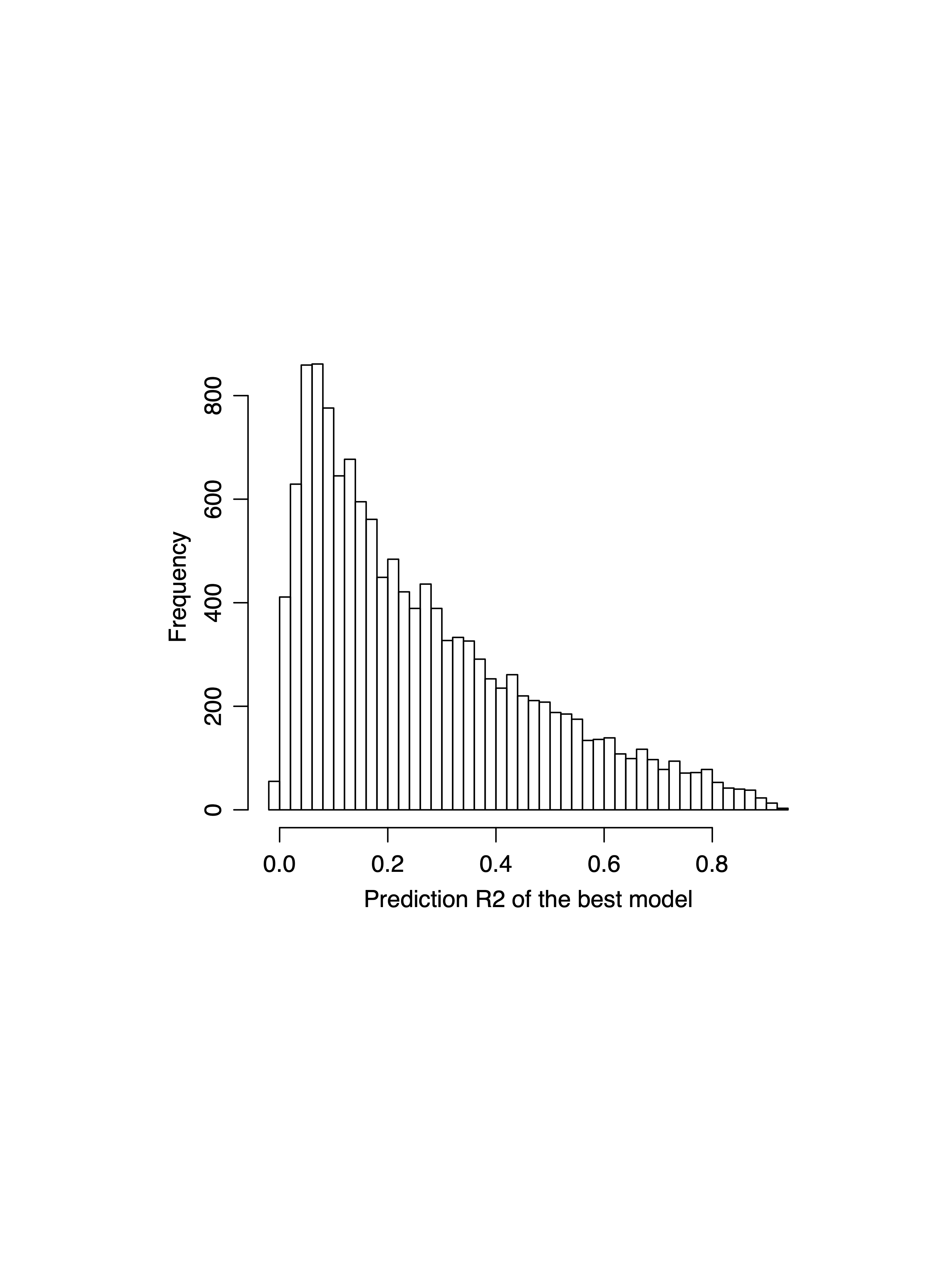

Supplement: S1 Fig — (TIFF) [file pgen.1010693.s002.tiff]

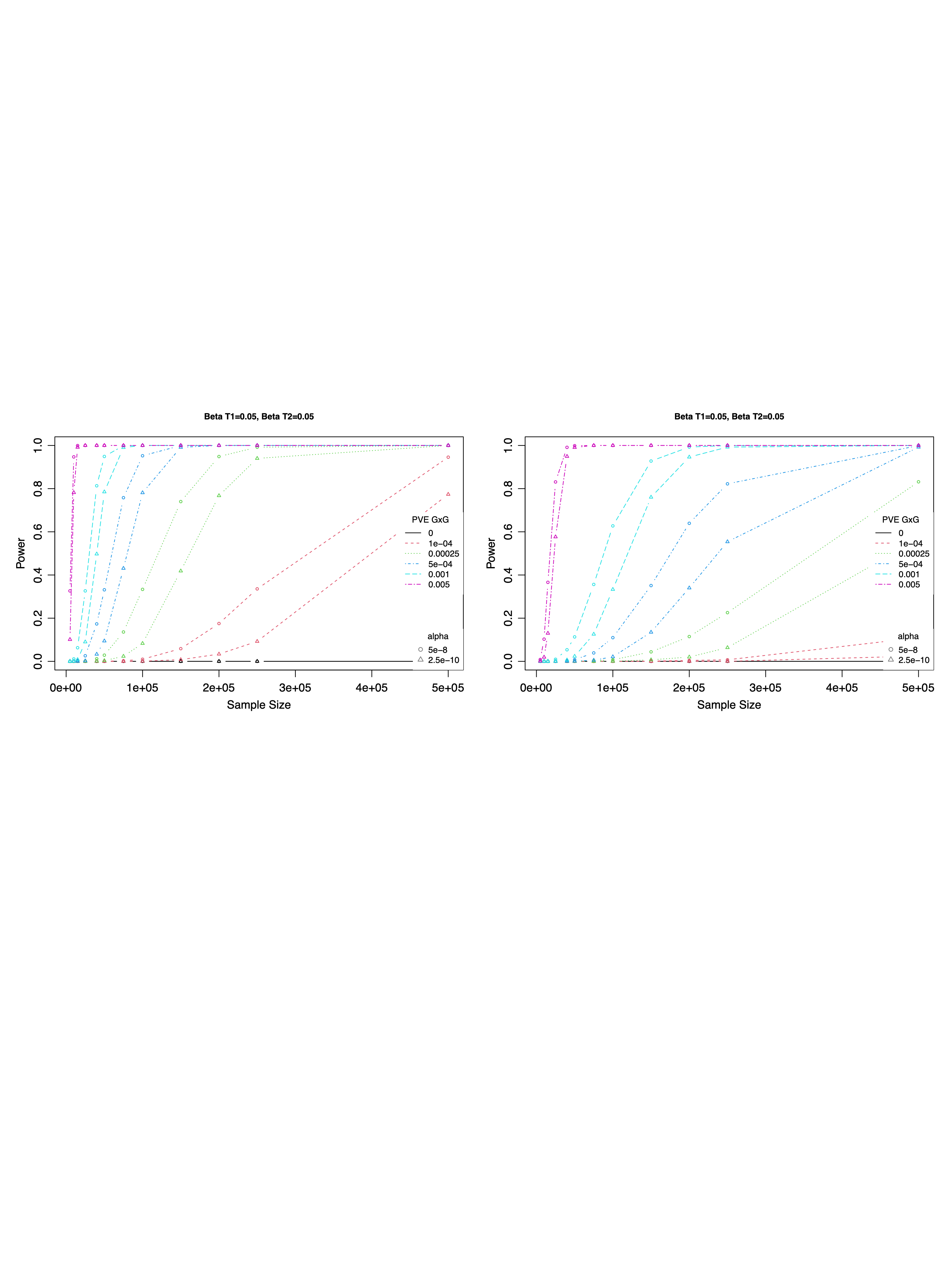

Supplement: S2 Fig — Left, without incorporating expression prediction error into the simulation. Right, incorporating random error for each predicted gene expression based on the distribution of observed prediction accuracies of the best model in S1 Fig. Main effect sizes for the two expression predictors (T1 & T2) are shown above each plot; varying these had minimal effect on the interaction test power. (TIFF) [file pgen.1010693.s003.tiff]

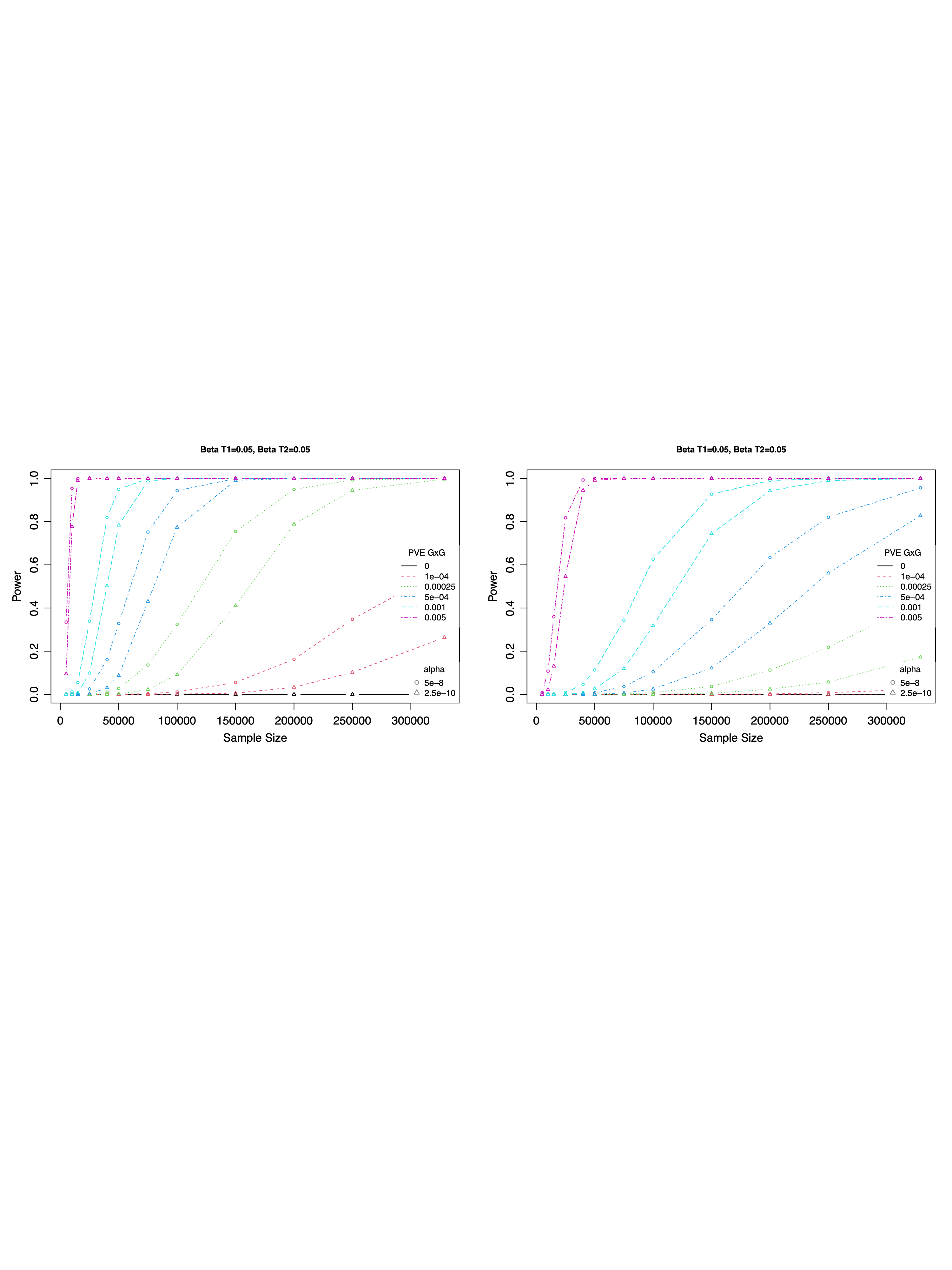

Supplement: S3 Fig — Left, without incorporating expression prediction error into the simulation. Right, incorporating random error for each predicted gene expression based on the observed prediction accuracies of the best model in S1 Fig. Main effect sizes for the two expression predictors (T1 & T2) are shown above each plot; varying these had minimal effect on the interaction test power. (TIFF) [file pgen.1010693.s004.tiff]

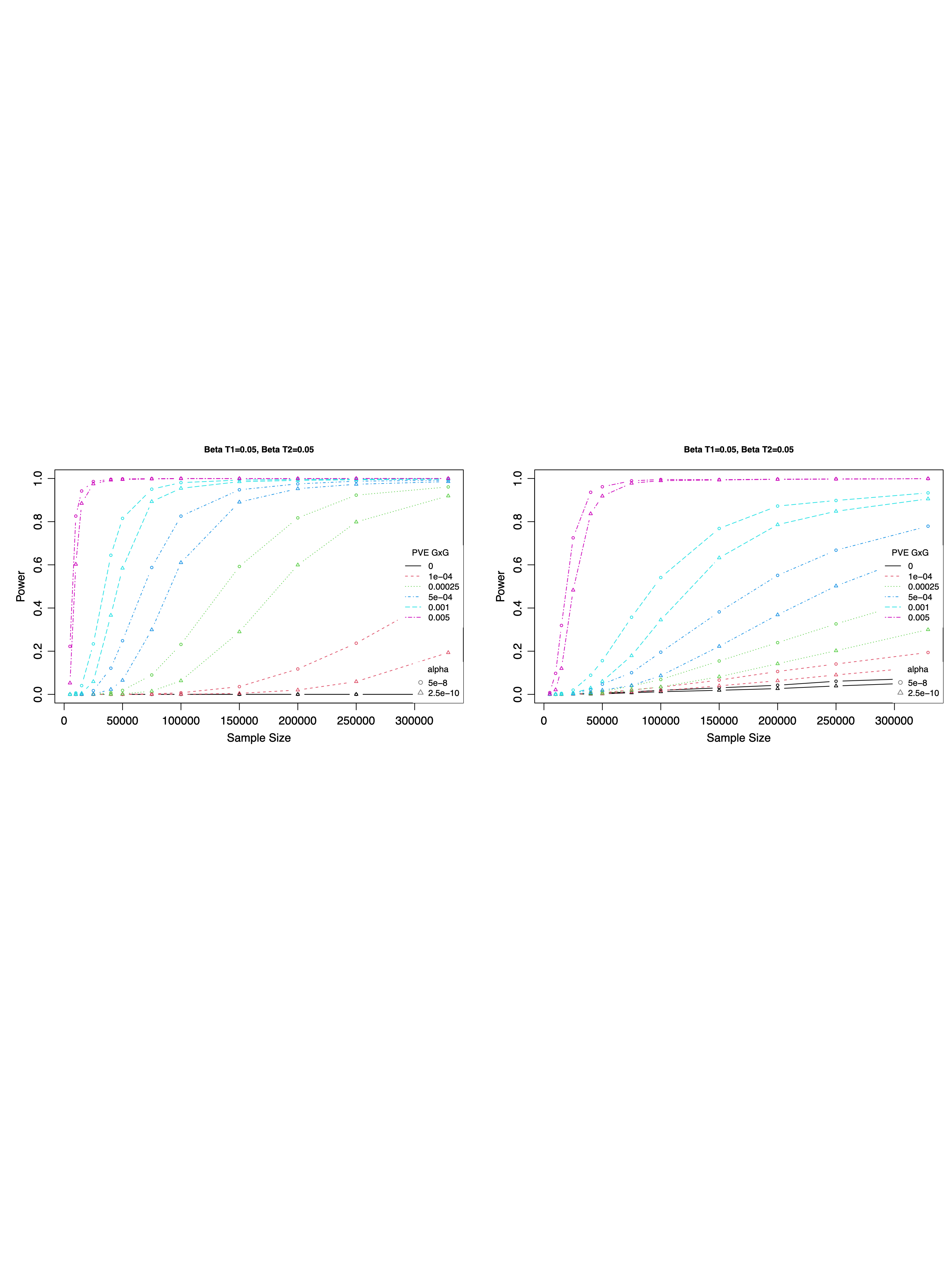

Supplement: S4 Fig — Left, without incorporating expression prediction error into the simulation. Right, incorporating random error for each predicted gene expression based on the observed prediction accuracies of the best model in S1 Fig. (TIFF) [file pgen.1010693.s005.tiff]

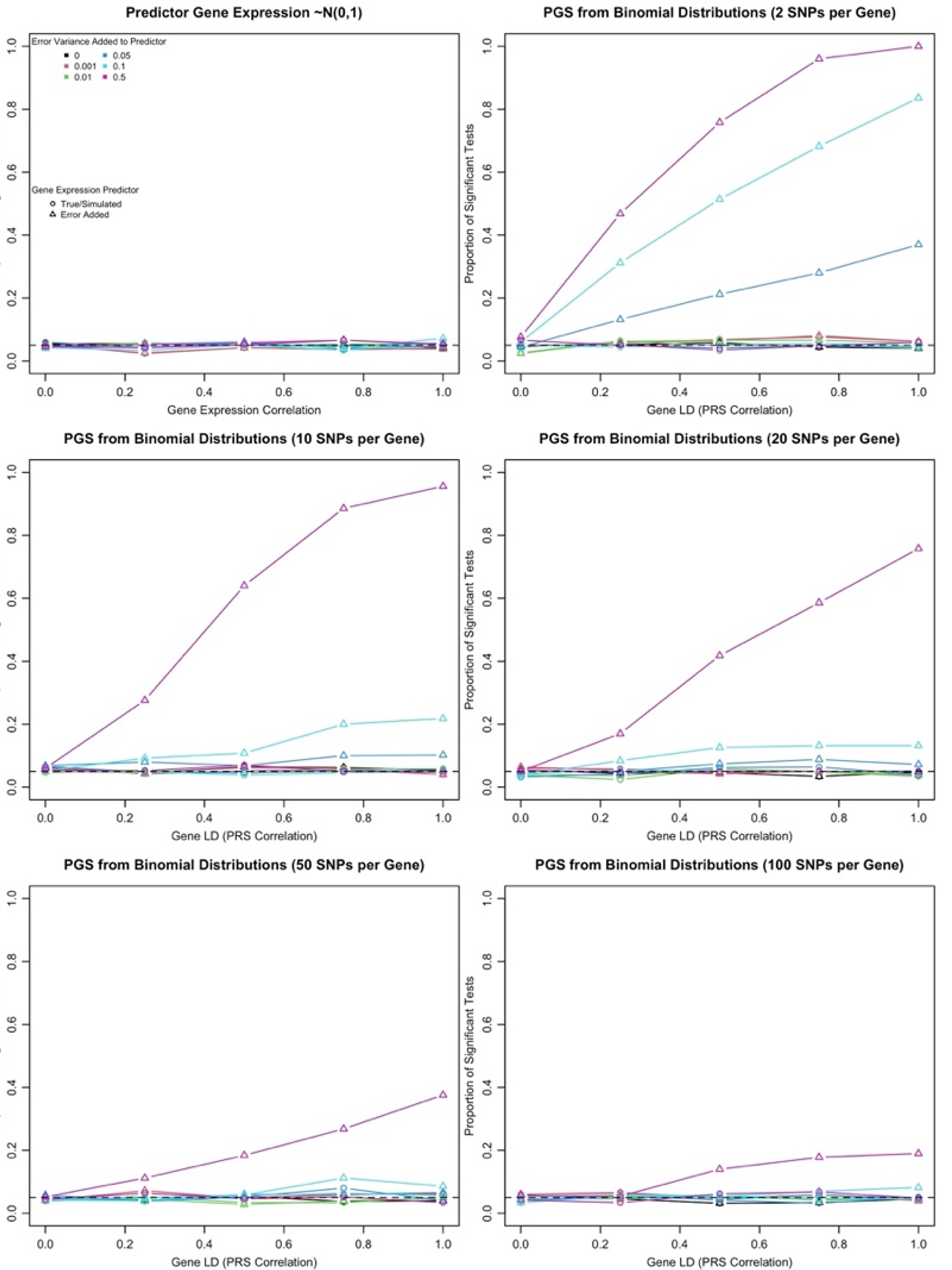

Supplement: S5 Fig — Simulated phenotypes included main effects of gene expression (based on varying polygenicity), but did not include gene expression interaction effects. When using truly normally distributed gene expression values in the regression (top), the test statistic is well calibrated (i.e., Type I error rate ~alpha), regardless of whether additional variance is added and whether estimated (i.e., imperfectly predicted) expression data are used. However, when the true expression data is generated from binomially distributed SNPs, using an imperfectly predicted PGS results in inflation of the Type I error rate, proportional to how poorly the PGS predicts expression, i.e., with increasing error variance added to the predictor. Note that this does not occur when the true observed expression is used, even if binomially distributed. The effect is greatest for a PGS using a single SNP, and weakens as the expression becomes more polygenic. (TIFF) [file pgen.1010693.s006.tiff]

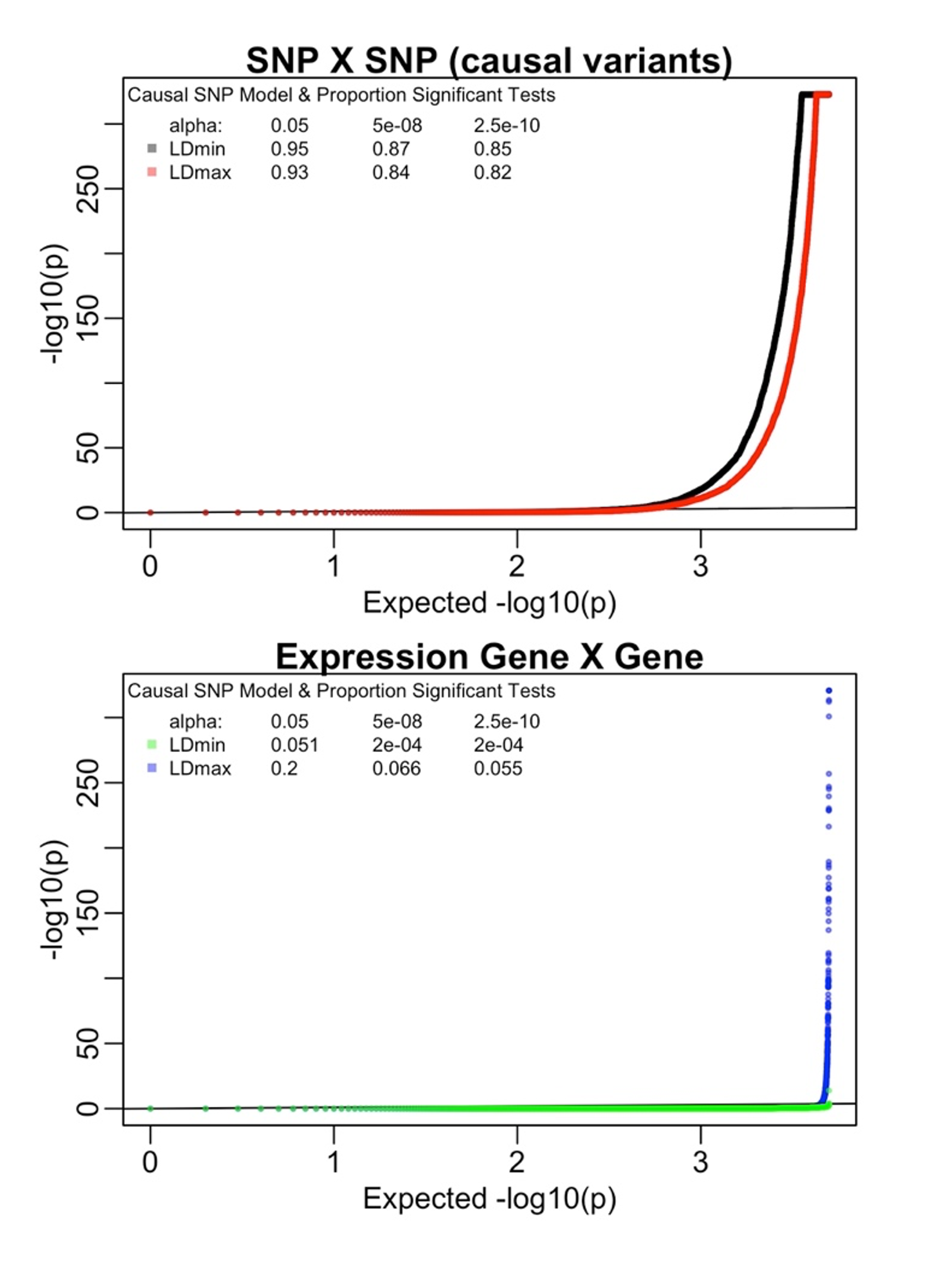

Supplement: S6 Fig — False positives increase when the SNPxSNP CVs have high LD scores than low LD scores, to the extent that the true effect is driven by SNP-SNP interactions, not expression-expression interactions. This results from LD between the causal SNPs and those used in the expression imputation. (TIFF) [file pgen.1010693.s007.tiff]

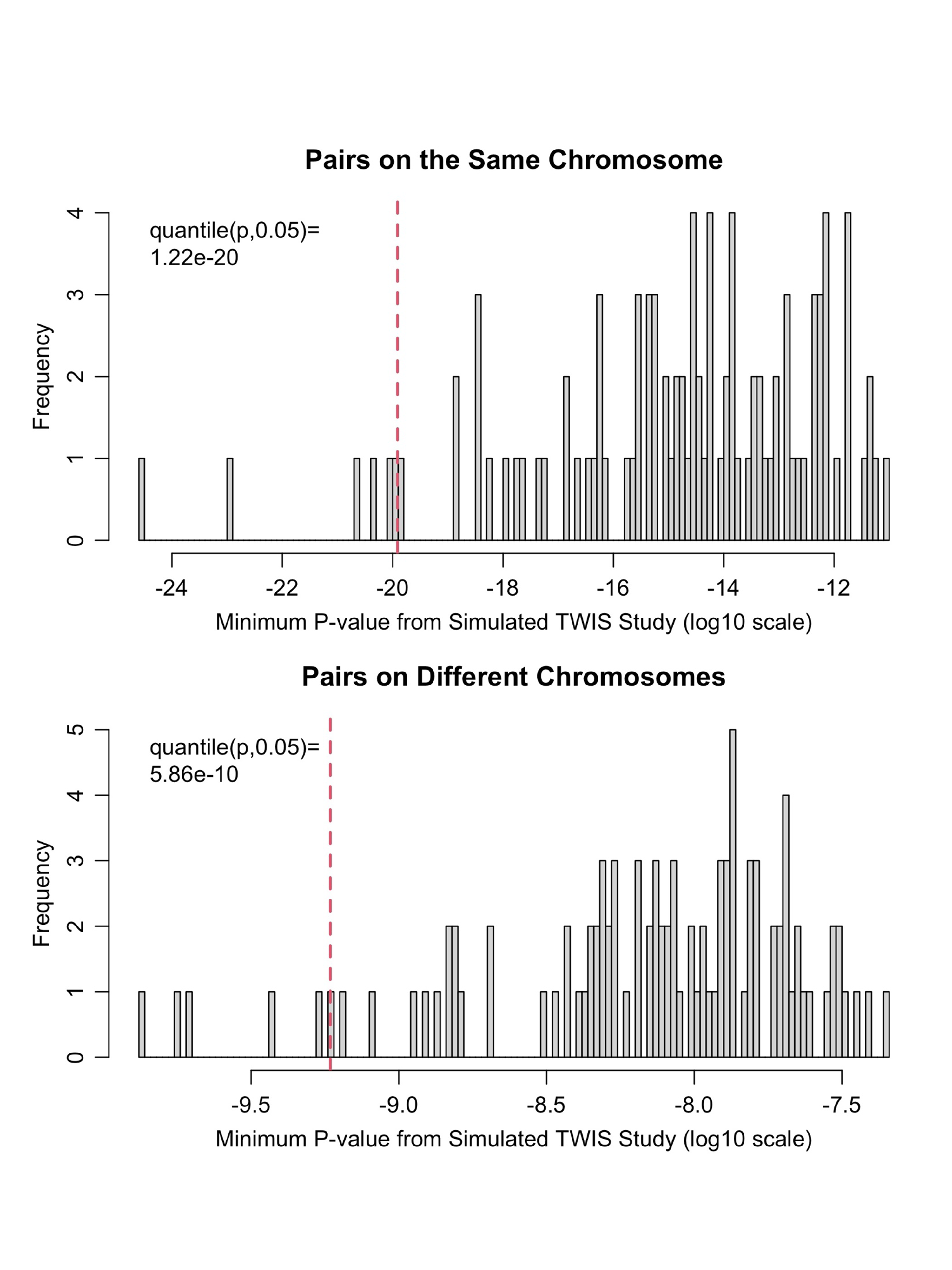

Supplement: S7 Fig — Red dashed line represents the 5th percentile of the minimum p-values. Note the x-axis scale differs between the two panels. (TIFF) [file pgen.1010693.s008.tiff]

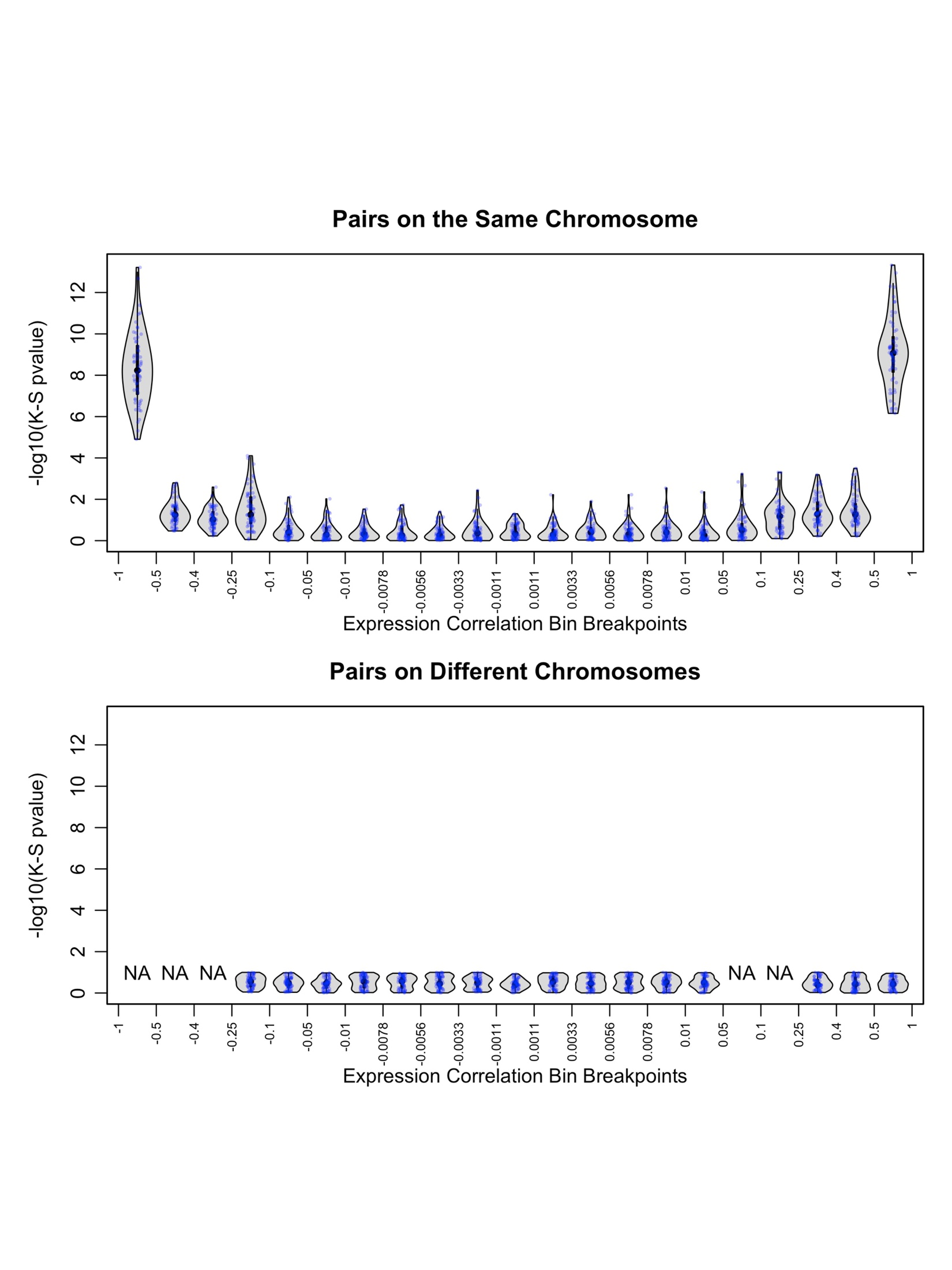

Supplement: S8 Fig — NA indicates no pairs of genes were found within that bin of pairwise imputed expression correlation. Blue dots are the (jittered) individual K-S test p-values for an entire simulated TWIS study. (TIFF) [file pgen.1010693.s009.tiff]

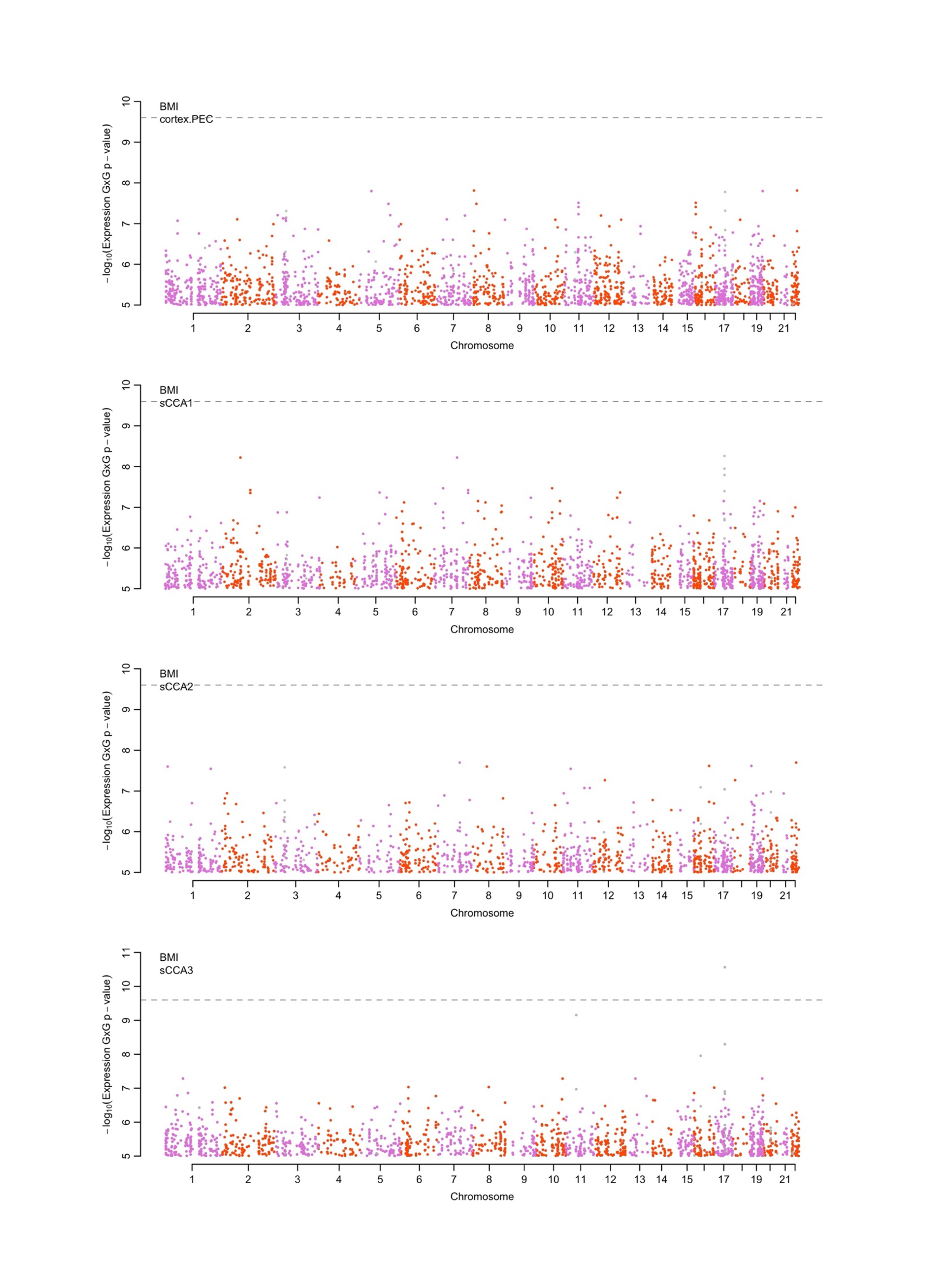

Supplement: S9 Fig — Shown are the results from the final meta-analysis of all data. Black lines connect pairs that surpassed p<2.5e-10 in the discovery cohort (UKB), blue lines connect pairs of loci with nominally significant interaction (p<0.05) in the replication cohort, and gray lines connect pairs of genes with p<2.5e-10 in the final meta-analysis. (TIFF) [file pgen.1010693.s010.tiff]

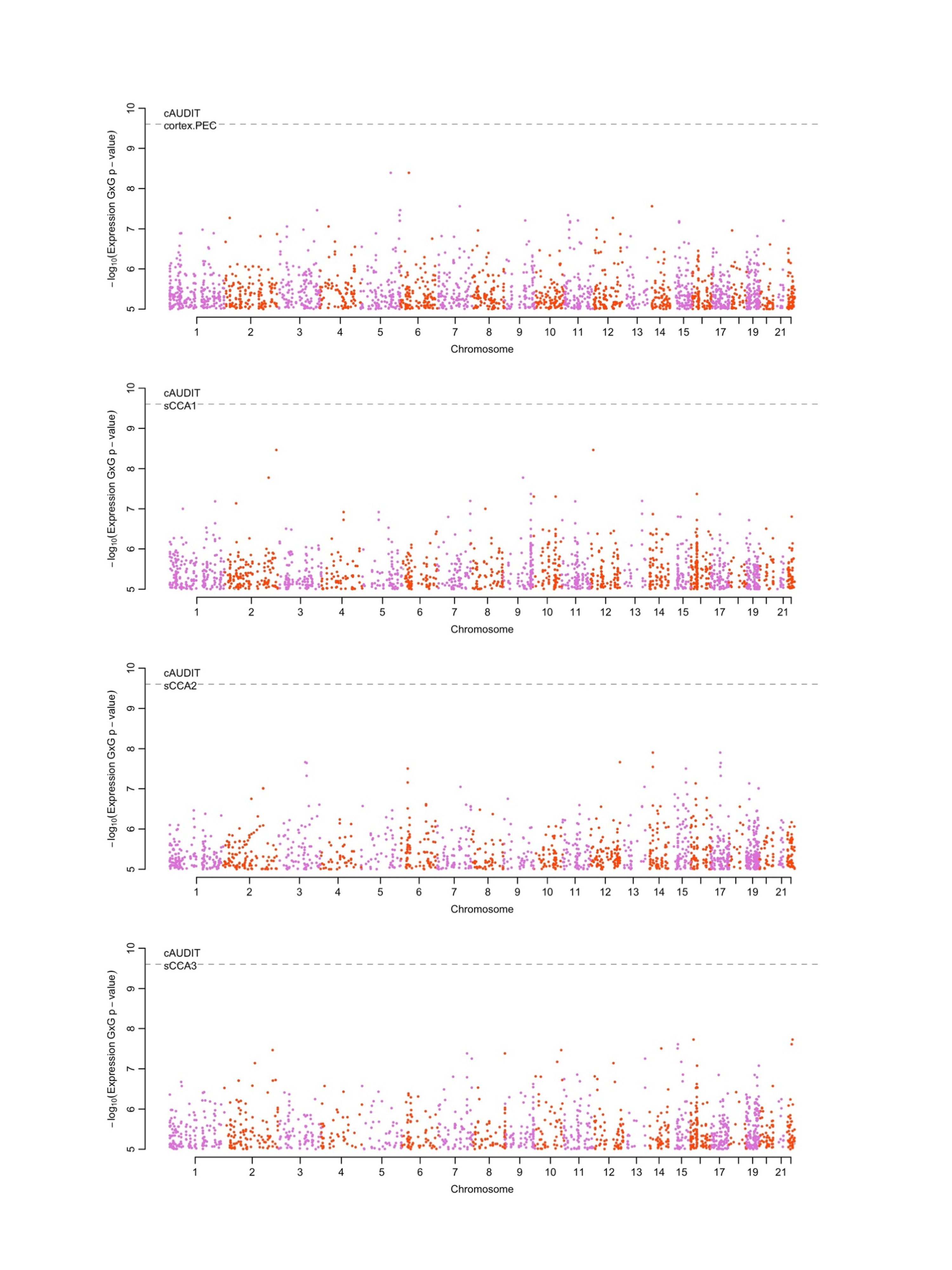

Supplement: S10 Fig — Shown are the results from the final meta-analysis of all data. Black lines connect pairs that surpassed p<2.5e-10 in the discovery cohort (UKB), blue lines connect pairs of loci with nominally significant interaction (p<0.05) in the replication cohort, and gray lines connect pairs of genes with p<2.5e-10 in the final meta-analysis. (TIFF) [file pgen.1010693.s011.tiff]

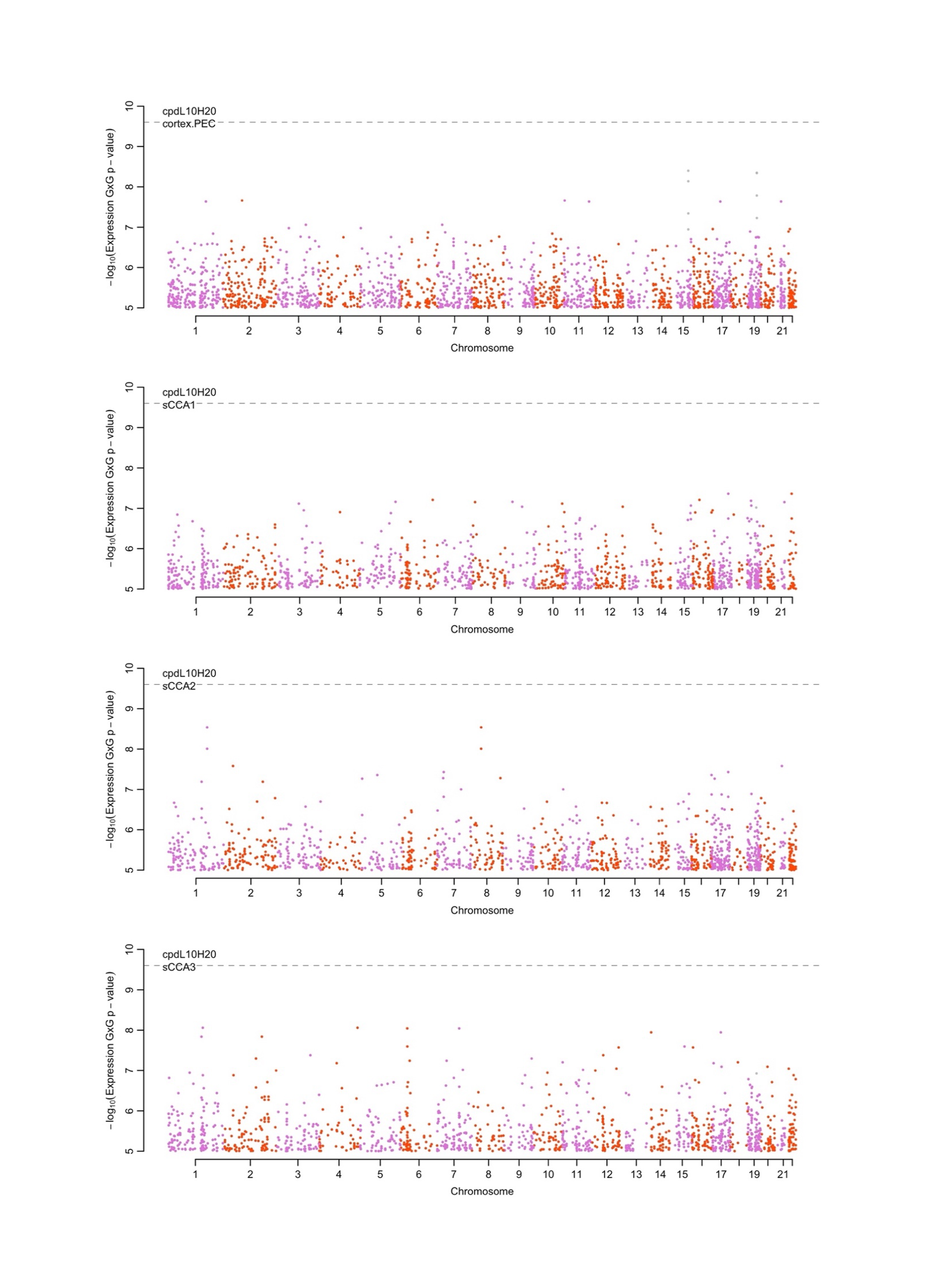

Supplement: S11 Fig — Shown are the results from the final meta-analysis of all data. Black lines connect pairs that surpassed p<2.5e-10 in the discovery cohort (UKB), blue lines connect pairs of loci with nominally significant interaction (p<0.05) in the replication cohort, and gray lines connect pairs of genes with p<2.5e-10 in the final meta-analysis. (TIFF) [file pgen.1010693.s012.tiff]

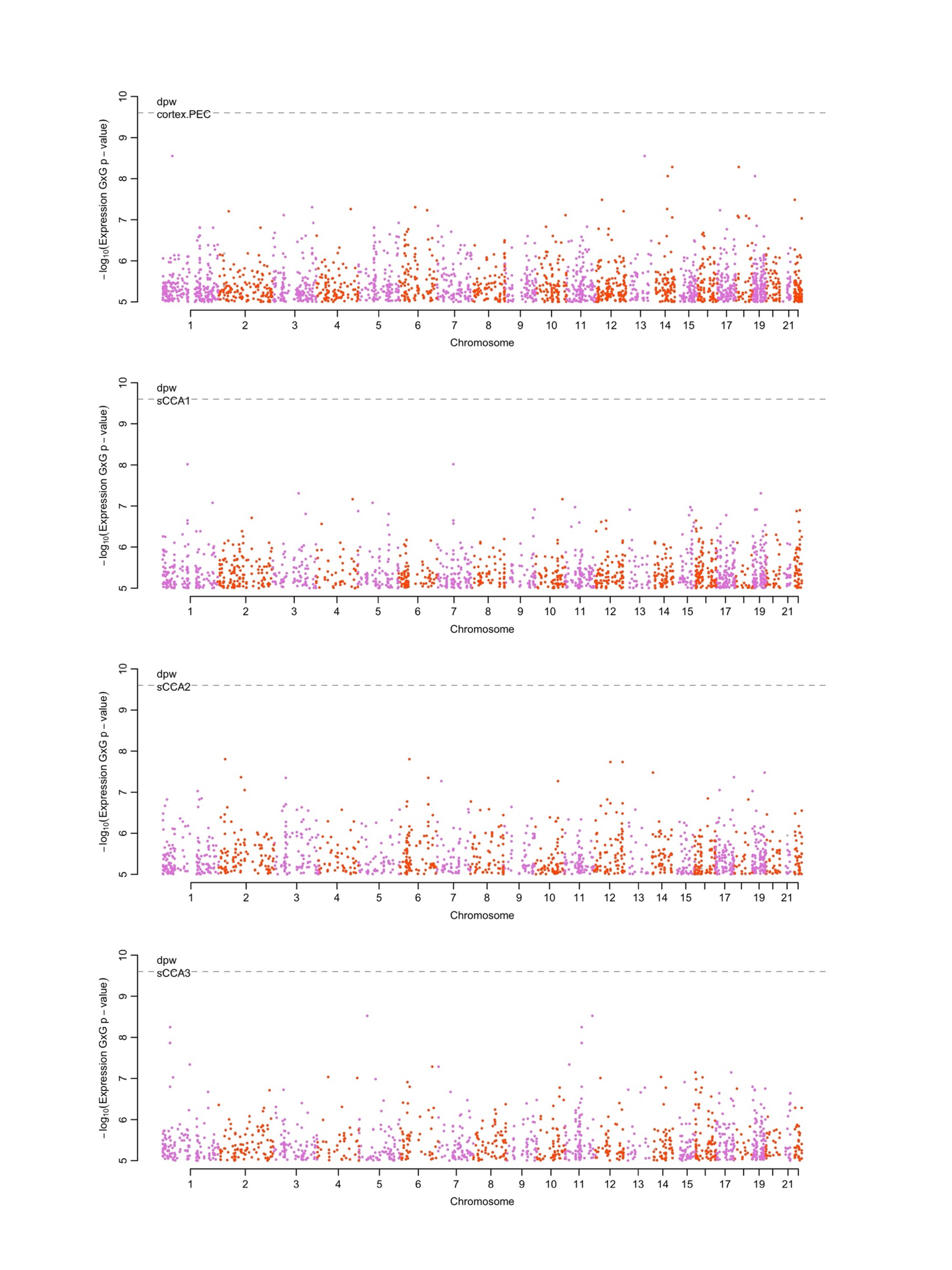

Supplement: S12 Fig — Shown are the results from the final meta-analysis of all data. Black lines connect pairs that surpassed p<2.5e-10 in the discovery cohort (UKB), blue lines connect pairs of loci with nominally significant interaction (p<0.05) in the replication cohort, and gray lines connect pairs of genes with p<2.5e-10 in the final meta-analysis. (TIFF) [file pgen.1010693.s013.tiff]

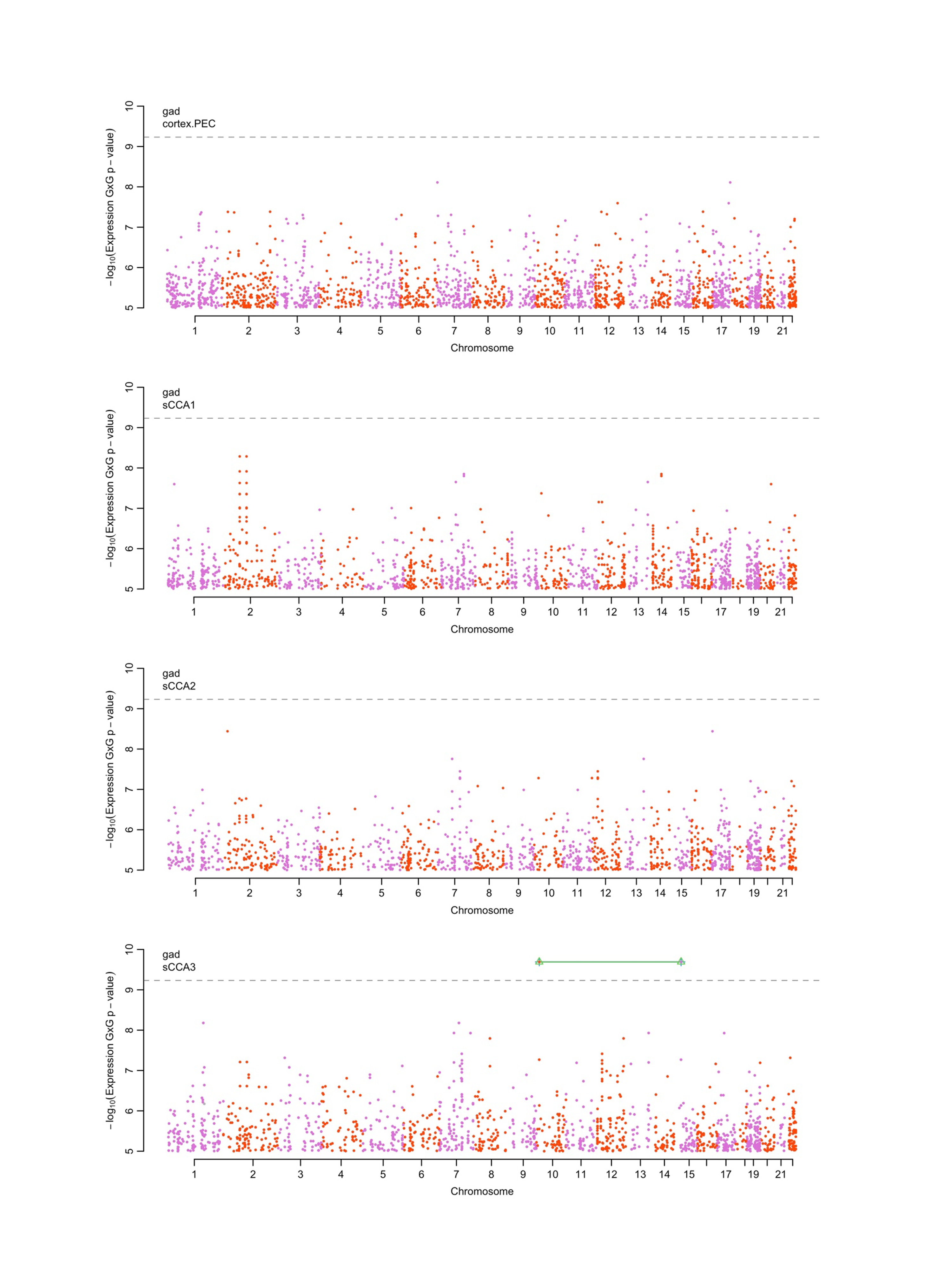

Supplement: S13 Fig — Shown are the results from the final meta-analysis of all data. Black lines connect pairs that surpassed p<2.5e-10 in the discovery cohort (UKB), green lines connect pairs of loci with significant (q<0.05) in the replication cohort, and gray lines connect pairs of genes with p<2.5e-10 in the final meta-analysis. (TIFF) [file pgen.1010693.s014.tiff]

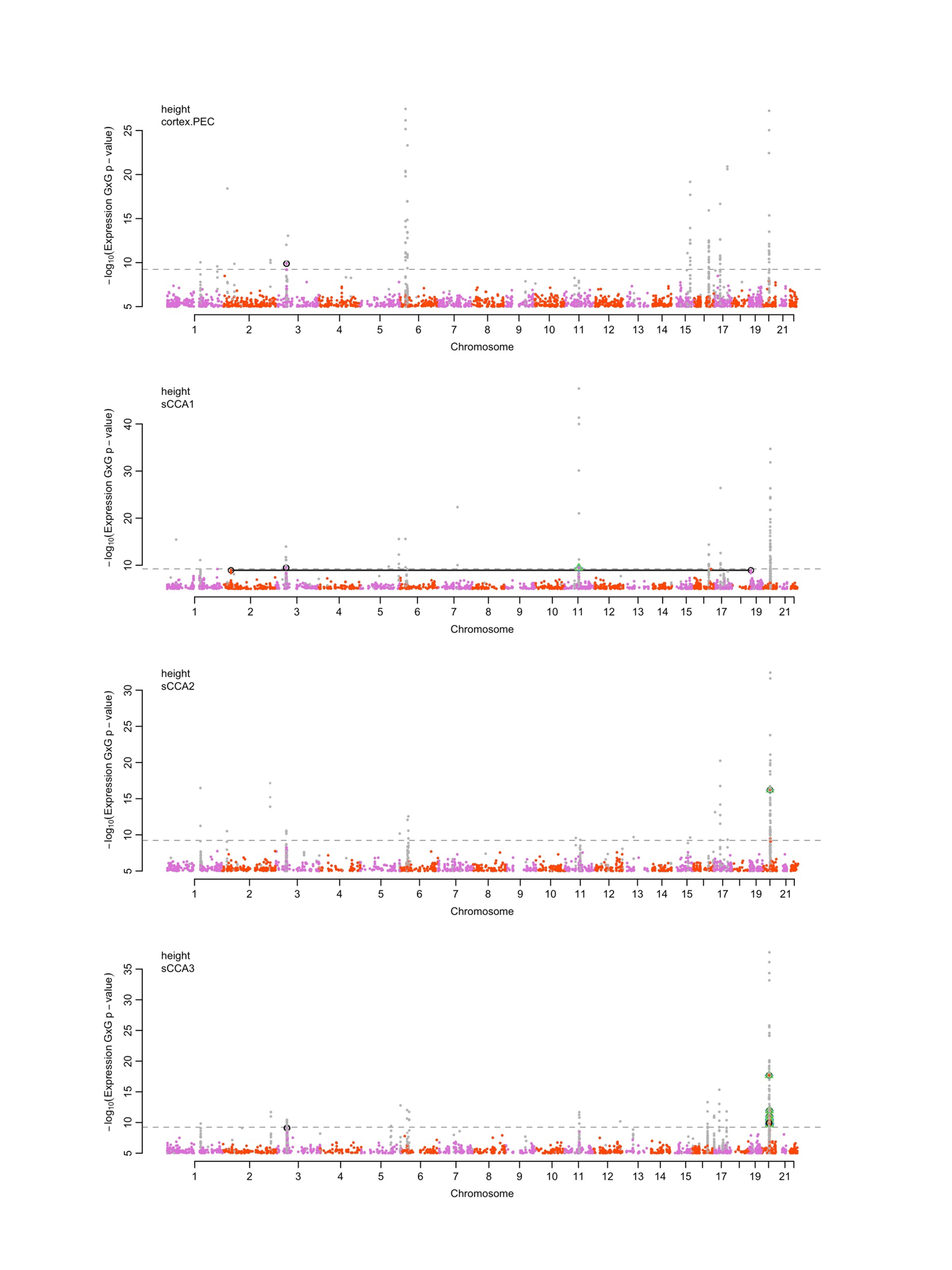

Supplement: S14 Fig — Shown are the results from the final meta-analysis of all data. Black lines connect pairs that surpassed p<2.5e-10 in the discovery cohort (UKB), blue lines connect pairs of loci with nominally significant interaction (p<0.05) in the replication cohort, and gray lines connect pairs of genes with p<2.5e-10 in the final meta-analysis. (TIFF) [file pgen.1010693.s015.tiff]

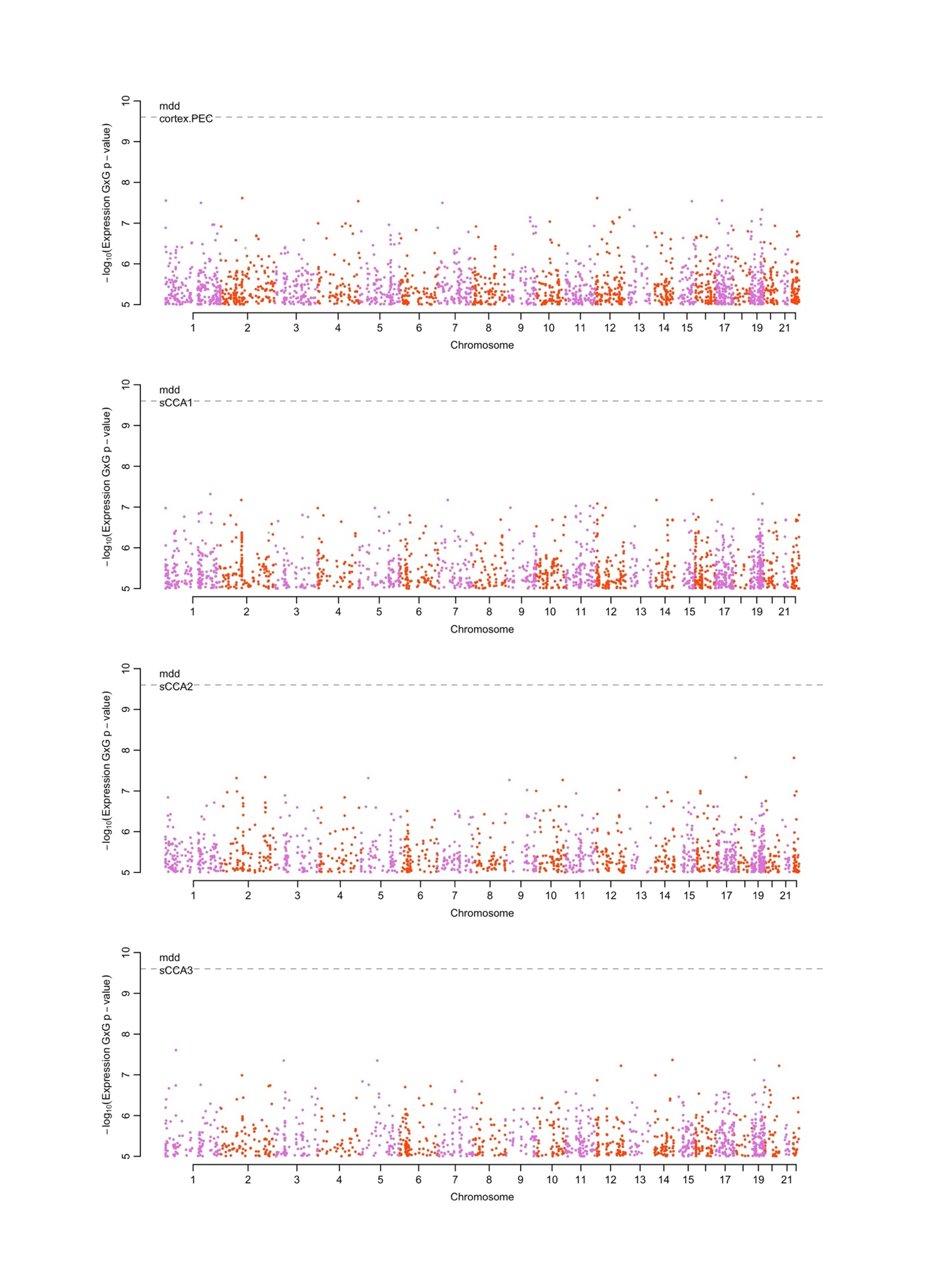

Supplement: S15 Fig — Shown are the results from the final meta-analysis of all data. Black lines connect pairs that surpassed p<2.5e-10 in the discovery cohort (UKB), blue lines connect pairs of loci with nominally significant interaction (p<0.05) in the replication cohort, and gray lines connect pairs of genes with p<2.5e-10 in the final meta-analysis. (TIFF) [file pgen.1010693.s016.tiff]

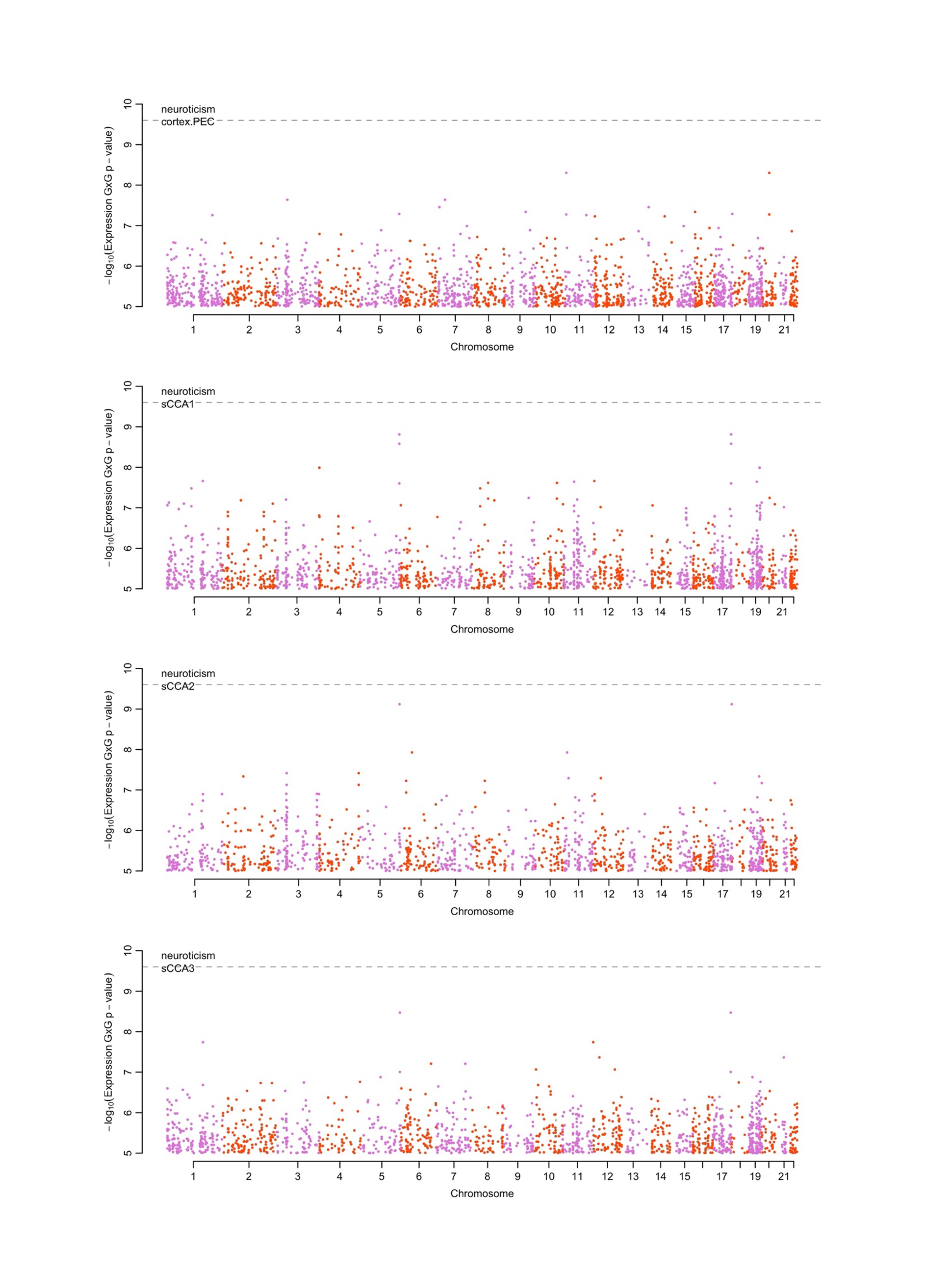

Supplement: S16 Fig — Shown are the results from the final meta-analysis of all data. Black lines connect pairs that surpassed p<2.5e-10 in the discovery cohort (UKB), blue lines connect pairs of loci with nominally significant interaction (p<0.05) in the replication cohort, and gray lines connect pairs of genes with p<2.5e-10 in the final meta-analysis. (TIFF) [file pgen.1010693.s017.tiff]

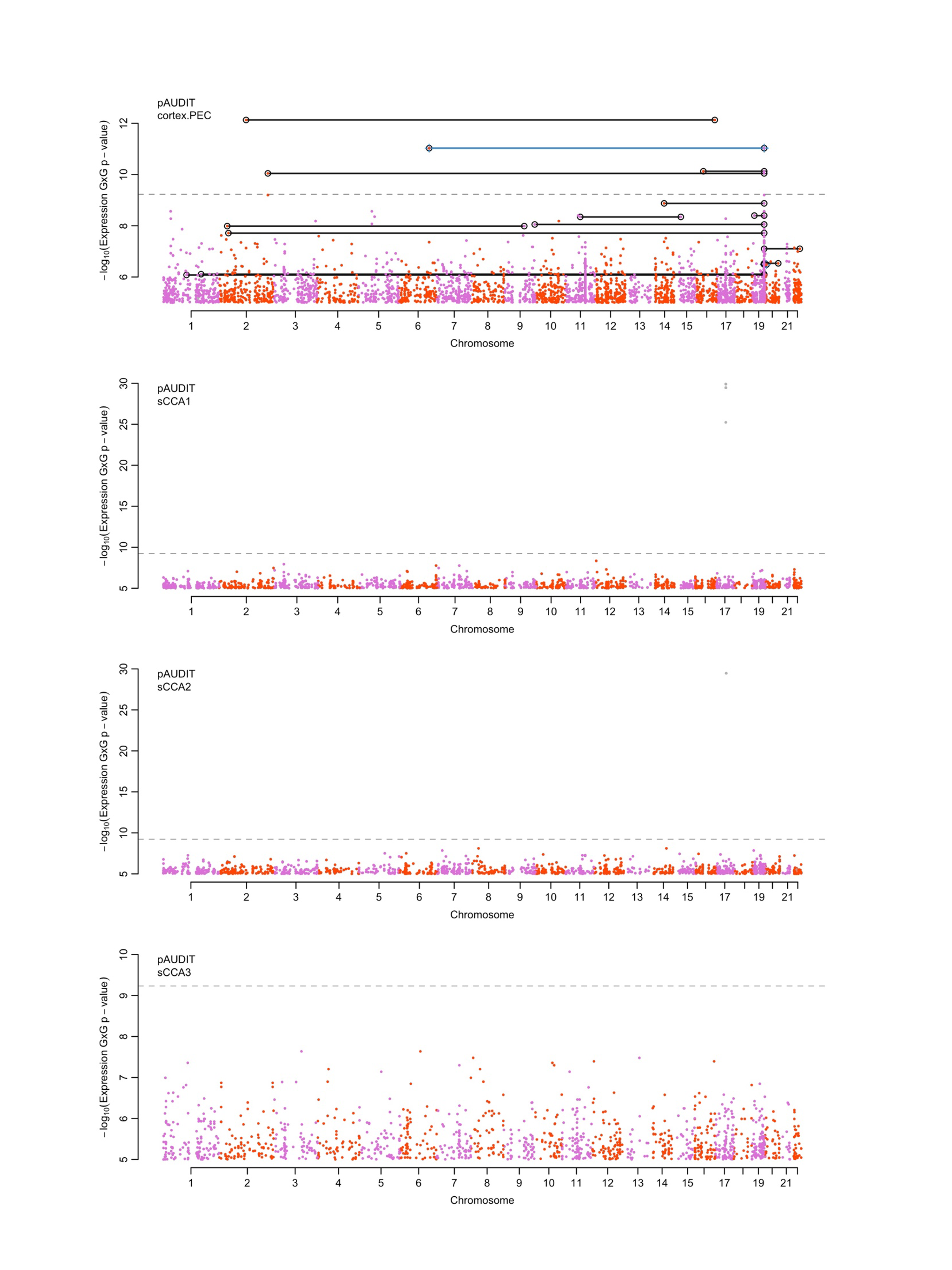

Supplement: S17 Fig — Shown are the results from the final meta-analysis of all data. Black lines connect pairs that surpassed p<2.5e-10 in the discovery cohort (UKB), blue lines connect pairs of loci with nominally significant interaction (p<0.05) in the replication cohort, and gray lines connect pairs of genes with p<2.5e-10 in the final meta-analysis. (TIFF) [file pgen.1010693.s018.tiff]

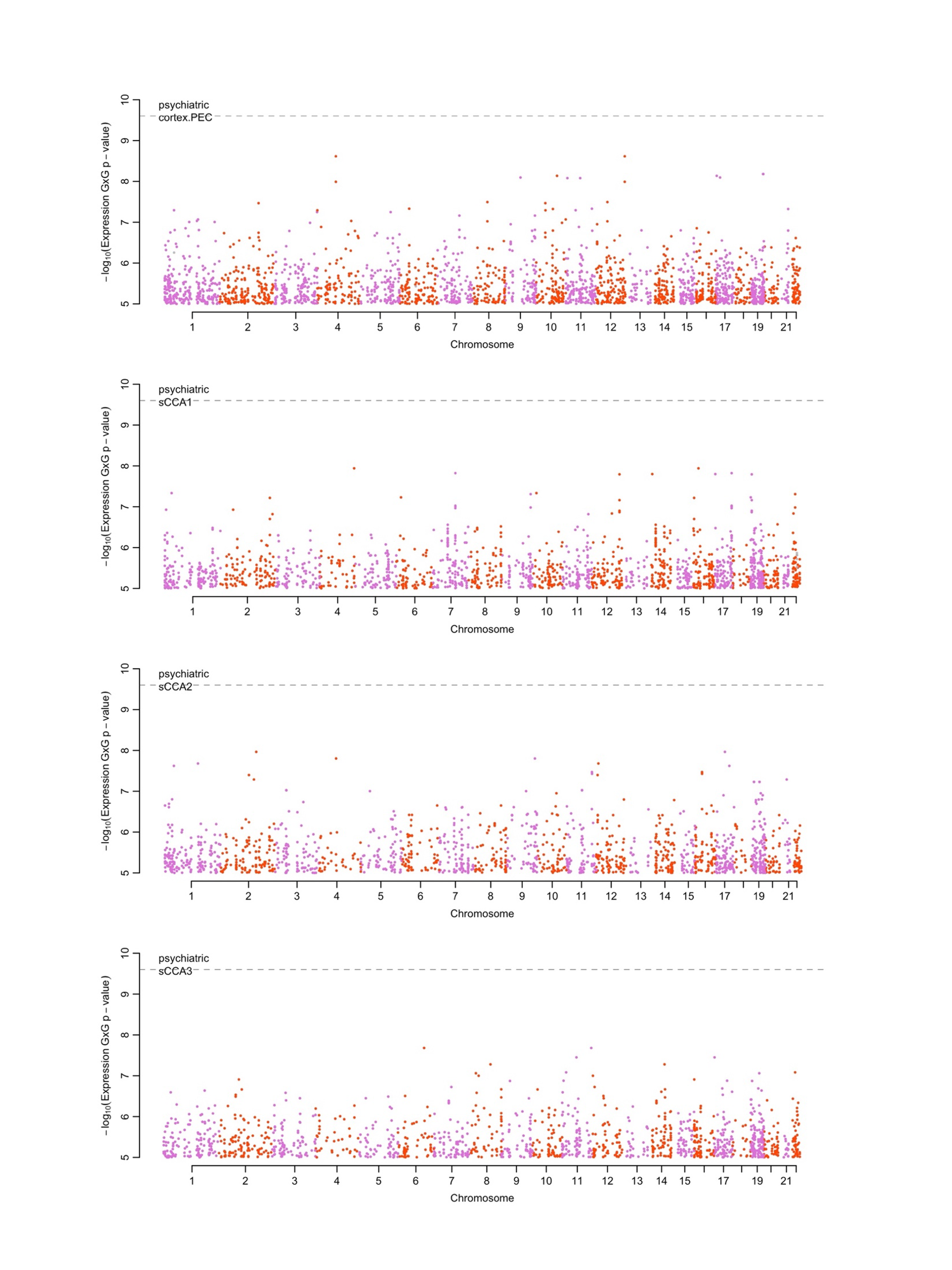

Supplement: S18 Fig — Shown are the results from the final meta-analysis of all data. Black lines connect pairs that surpassed p<2.5e-10 in the discovery cohort (UKB), blue lines connect pairs of loci with nominally significant interaction (p<0.05) in the replication cohort, and gray lines connect pairs of genes with p<2.5e-10 in the final meta-analysis. (TIFF) [file pgen.1010693.s019.tiff]

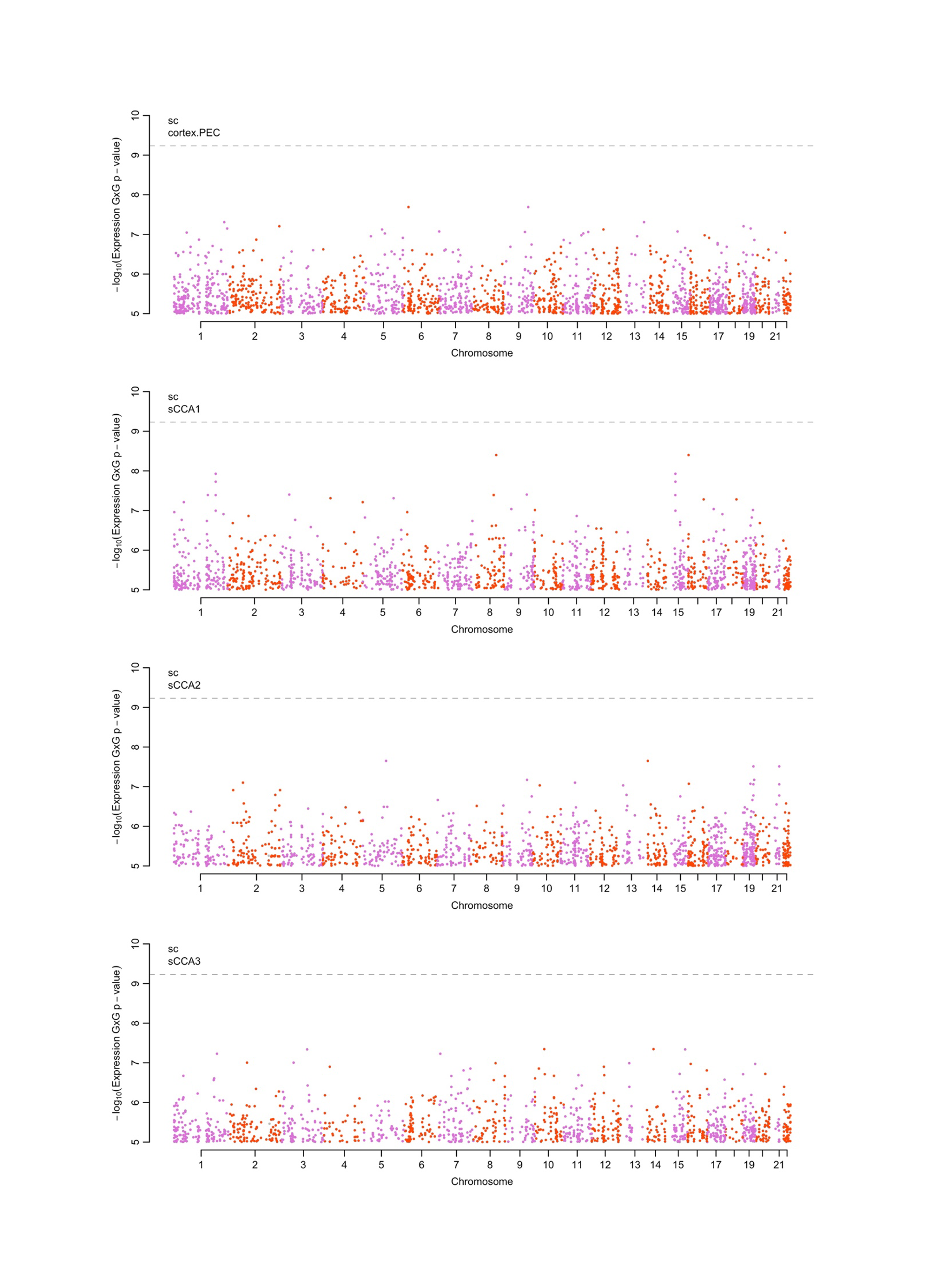

Supplement: S19 Fig — Shown are the results from the final meta-analysis of all data. Black lines connect pairs that surpassed p<2.5e-10 in the discovery cohort (UKB), blue lines connect pairs of loci with nominally significant interaction (p<0.05) in the replication cohort, and gray lines connect pairs of genes with p<2.5e-10 in the final meta-analysis. (TIFF) [file pgen.1010693.s020.tiff]

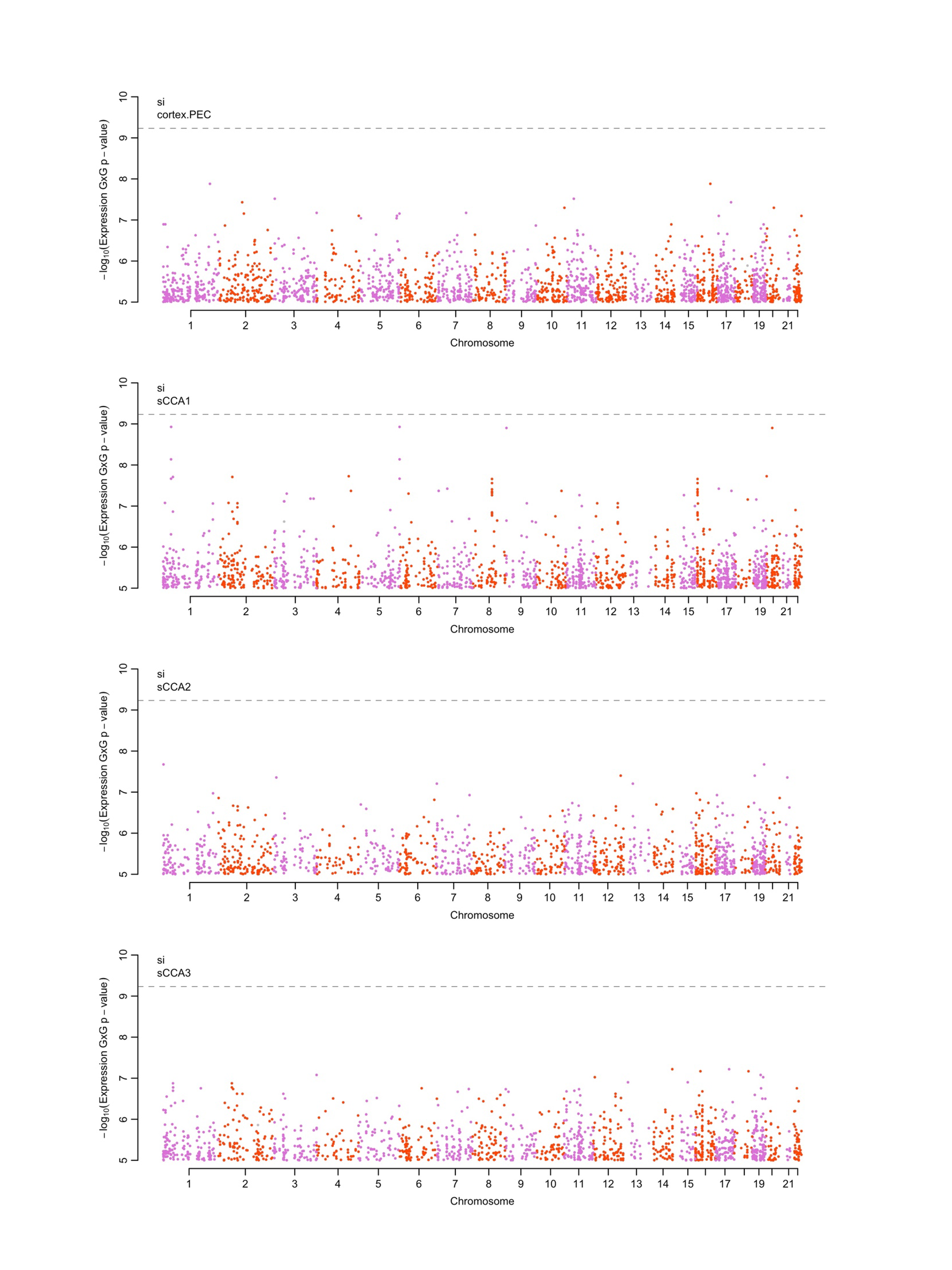

Supplement: S20 Fig — Shown are the results from the final meta-analysis of all data. Black lines connect pairs that surpassed p<2.5e-10 in the discovery cohort (UKB), blue lines connect pairs of loci with nominally significant interaction (p<0.05) in the replication cohort, and gray lines connect pairs of genes with p<2.5e-10 in the final meta-analysis. (TIFF) [file pgen.1010693.s021.tiff]

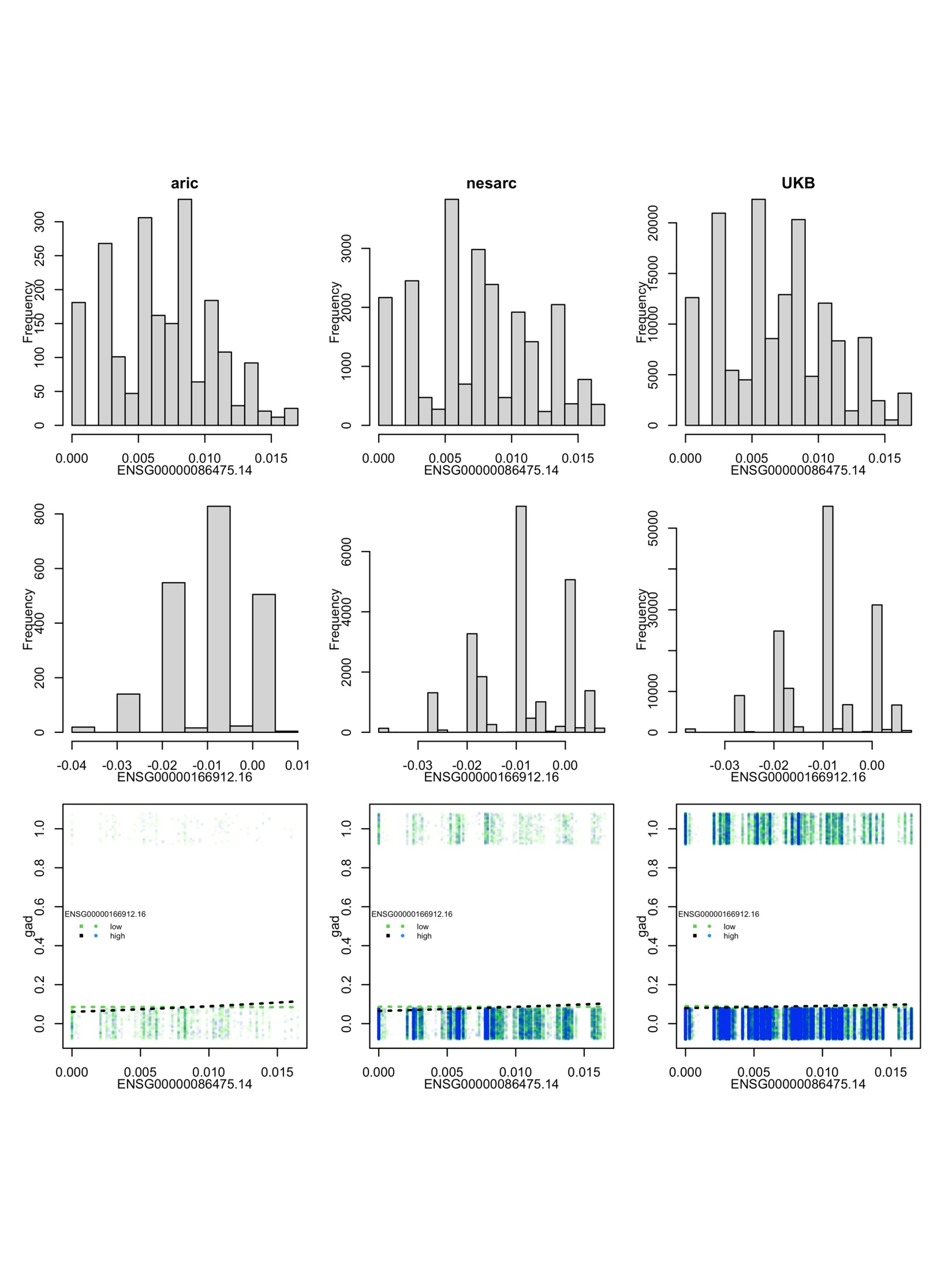

Supplement: S21 Fig — Studies are indicated in title of each panel. Fitted logistic regressions are shown by dashed line. (TIFF) [file pgen.1010693.s022.tiff]

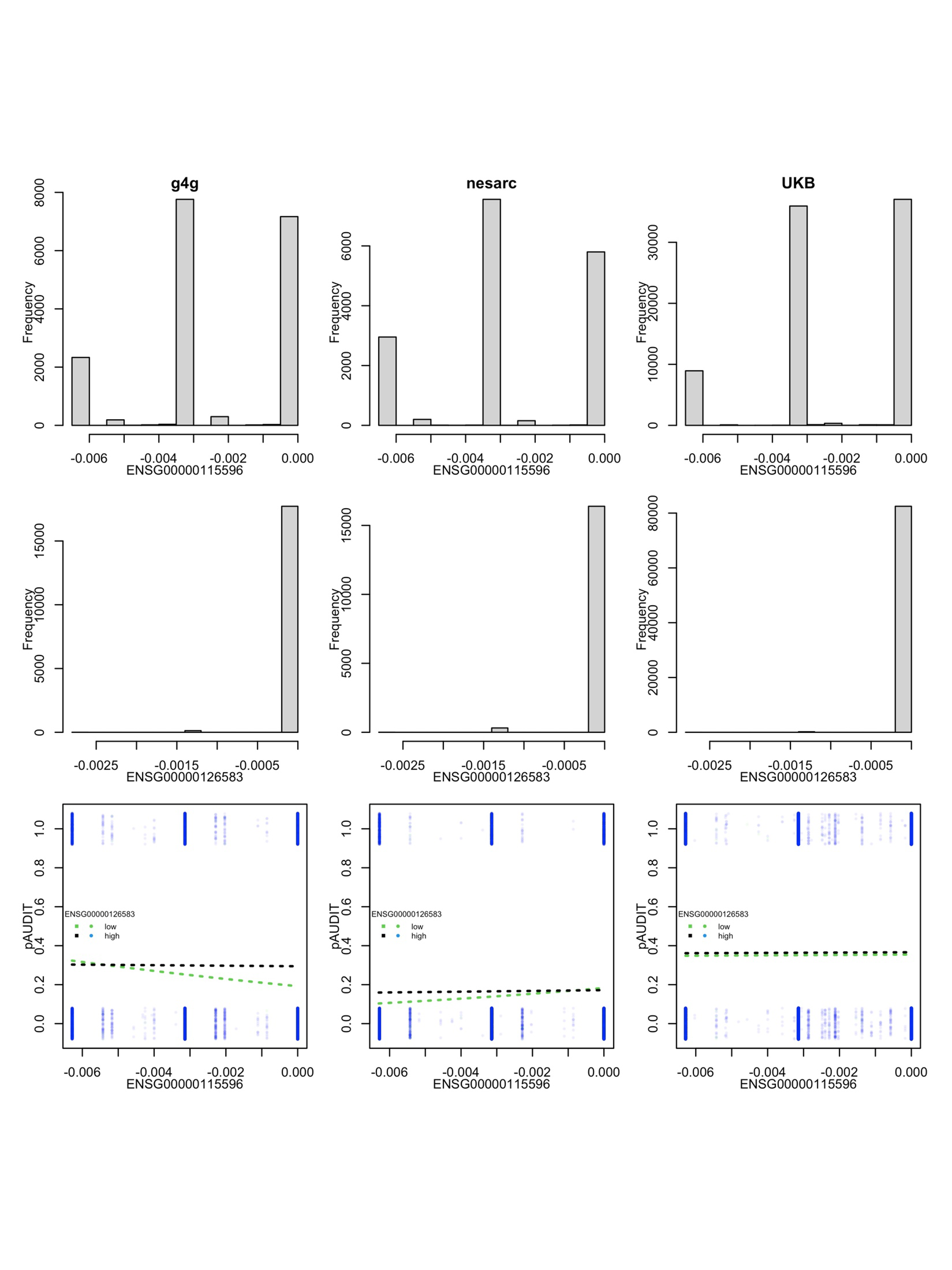

Supplement: S22 Fig — Studies are indicated in title of each panel. Fitted logistic regressions are shown by dashed line. (TIFF) [file pgen.1010693.s023.tiff]

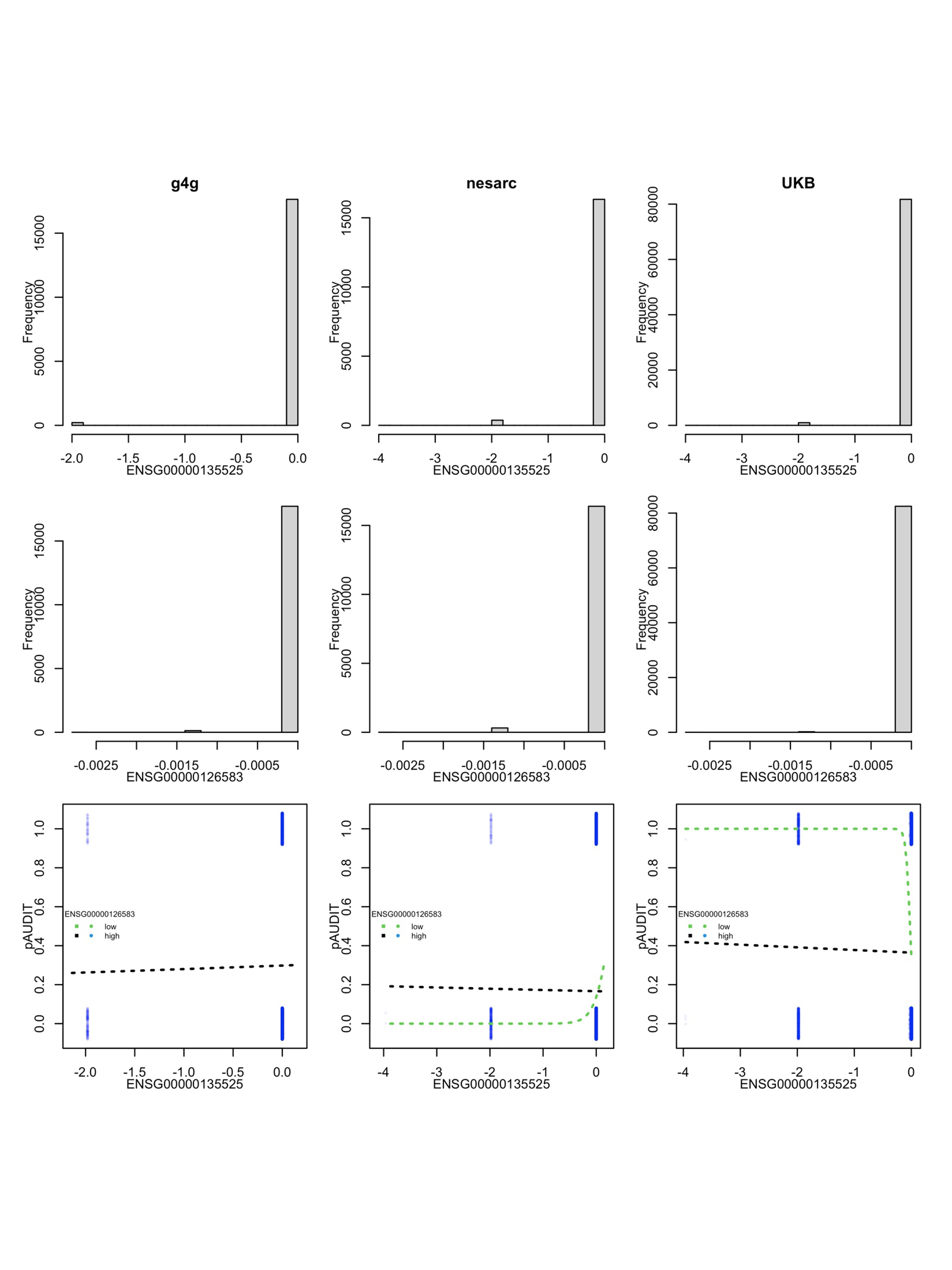

Supplement: S23 Fig — Studies are indicated in title of each panel. Fitted logistic regressions are shown by dashed line. (TIFF) [file pgen.1010693.s024.tiff]

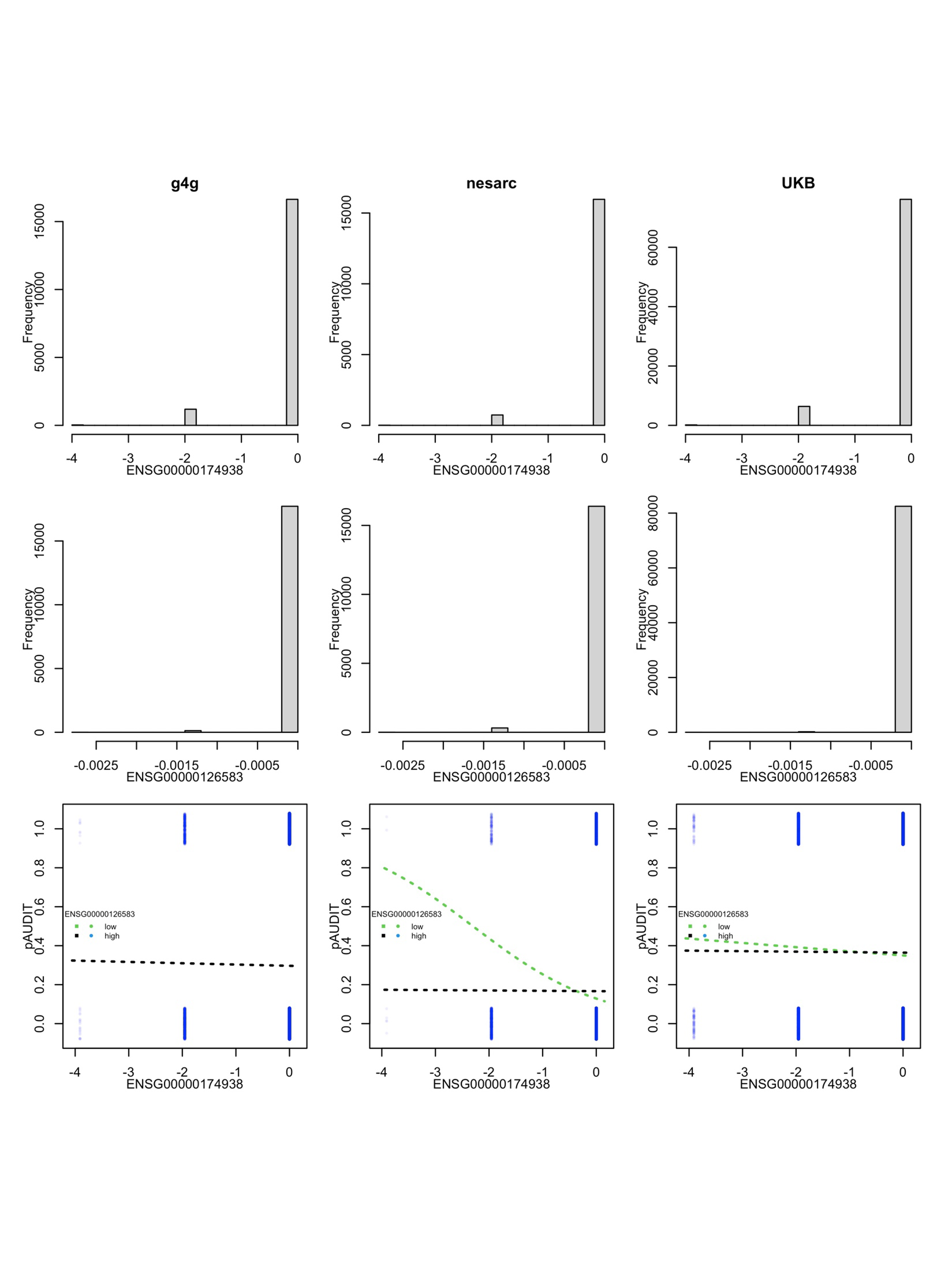

Supplement: S24 Fig — Studies are indicated in title of each panel. Fitted logistic regressions are shown by dashed line. (TIFF) [file pgen.1010693.s025.tiff]

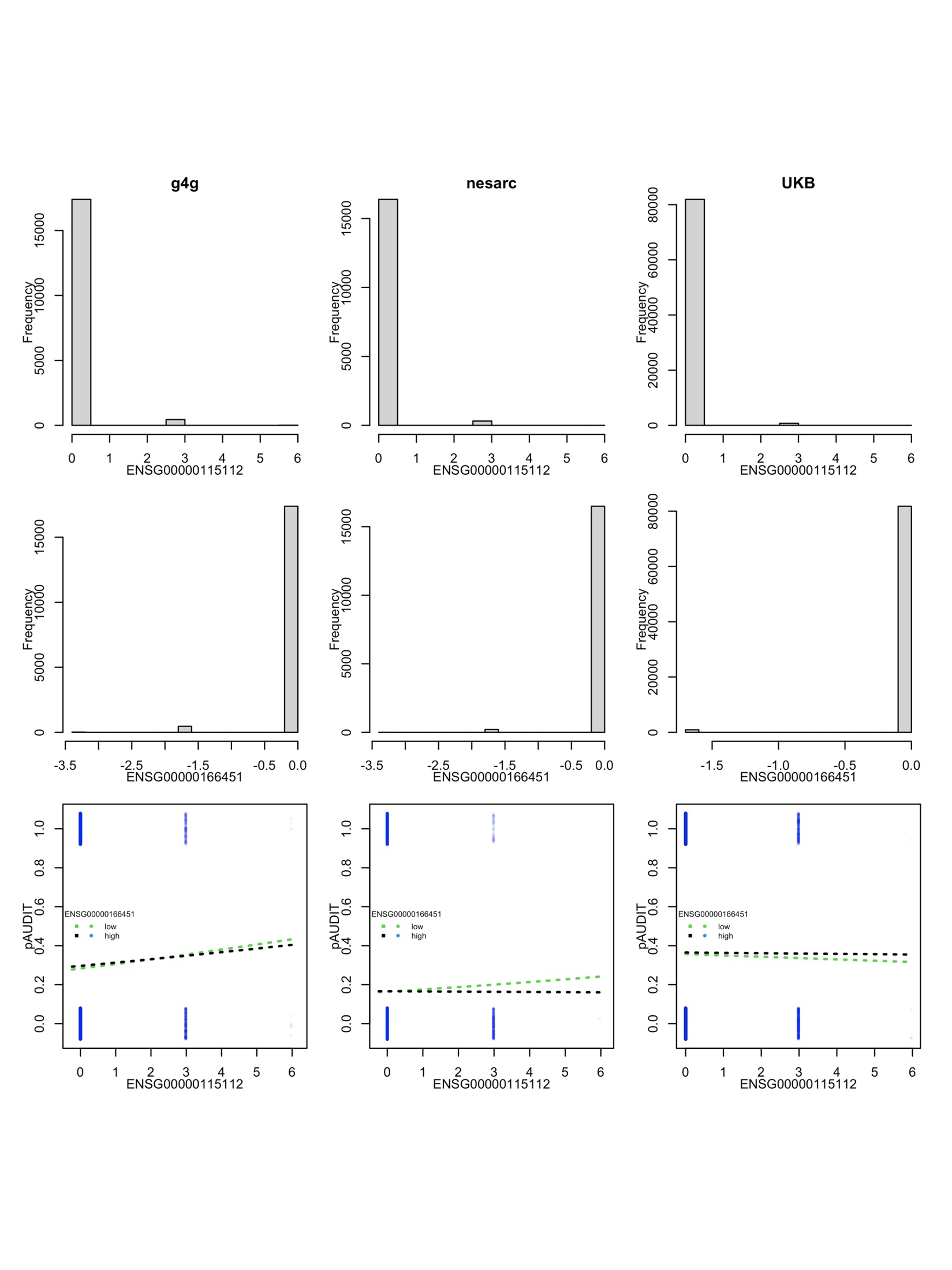

Supplement: S25 Fig — Studies are indicated in title of each panel. Fitted logistic regressions are shown by dashed line. (TIFF) [file pgen.1010693.s026.tiff]

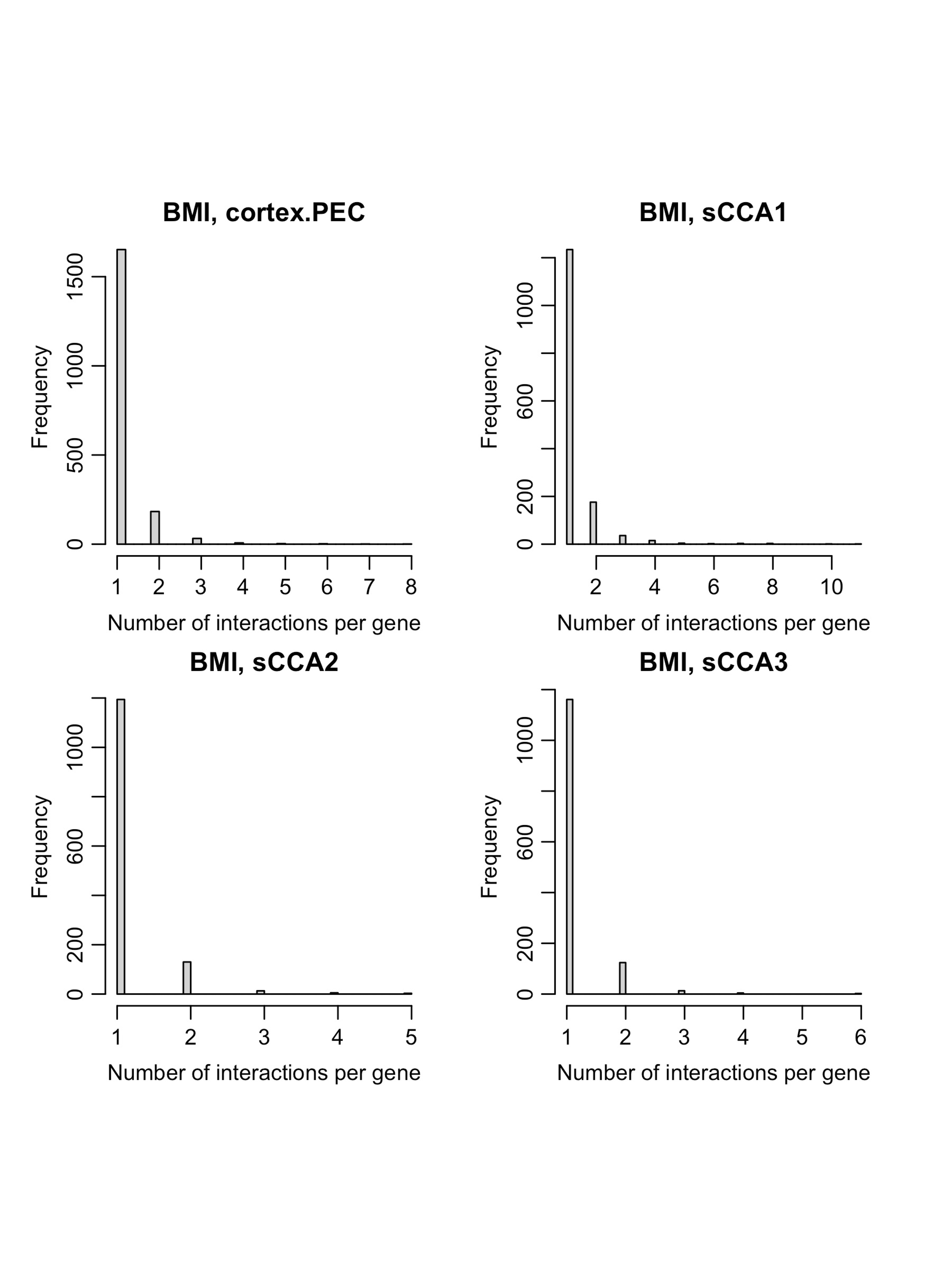

Supplement: S26 Fig — (TIFF) [file pgen.1010693.s027.tiff]

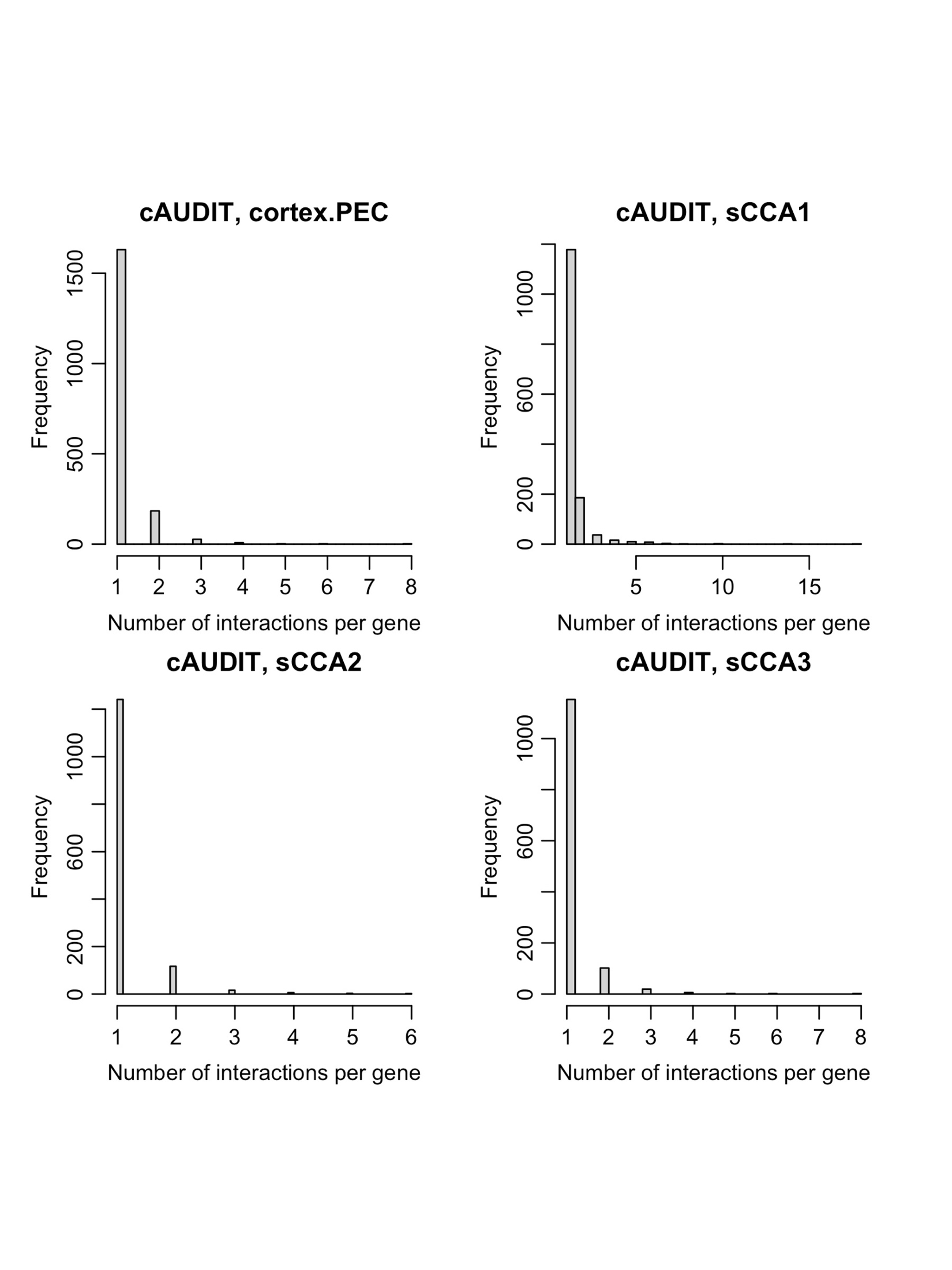

Supplement: S27 Fig — (TIFF) [file pgen.1010693.s028.tiff]

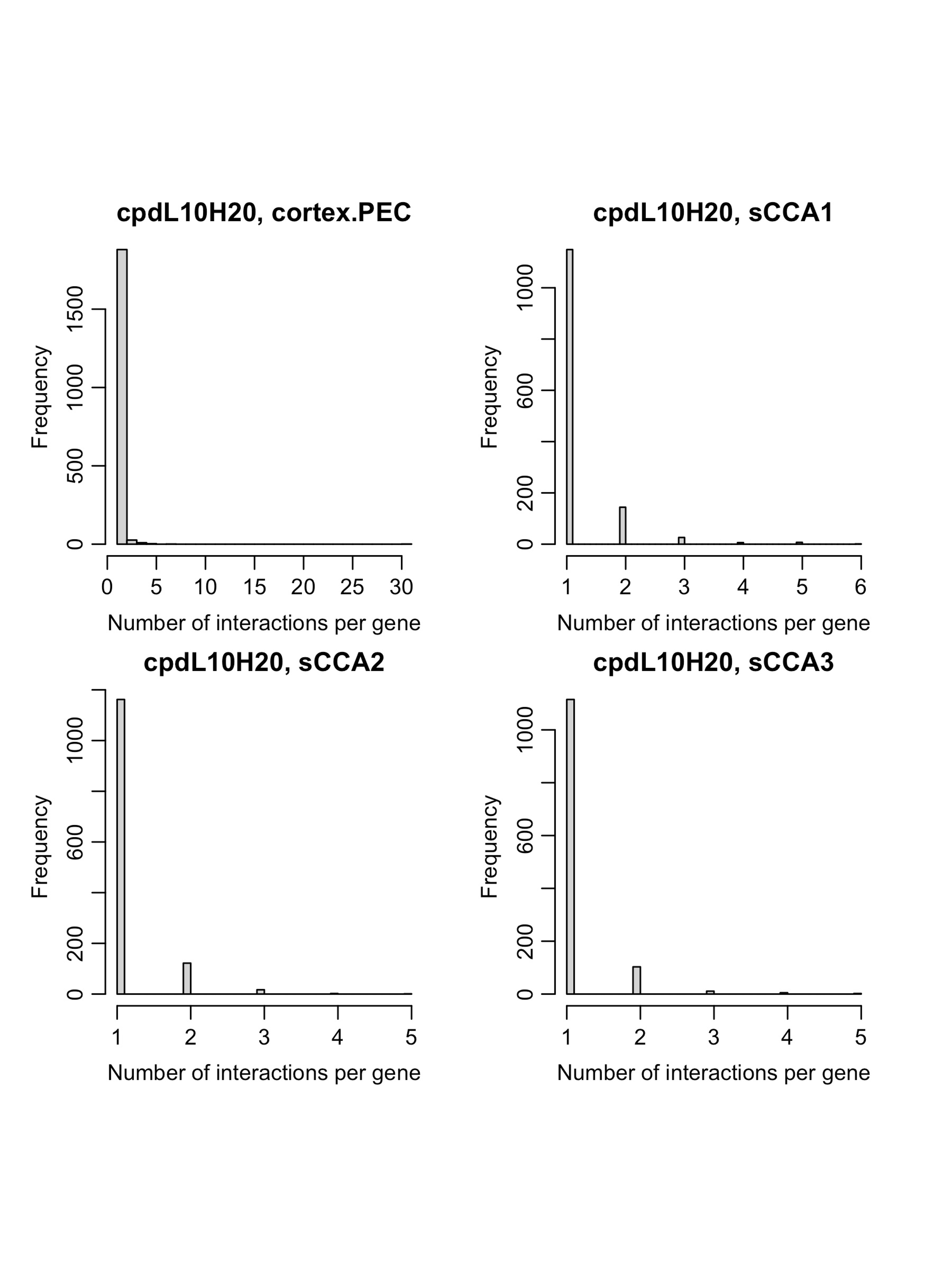

Supplement: S28 Fig — (TIFF) [file pgen.1010693.s029.tiff]

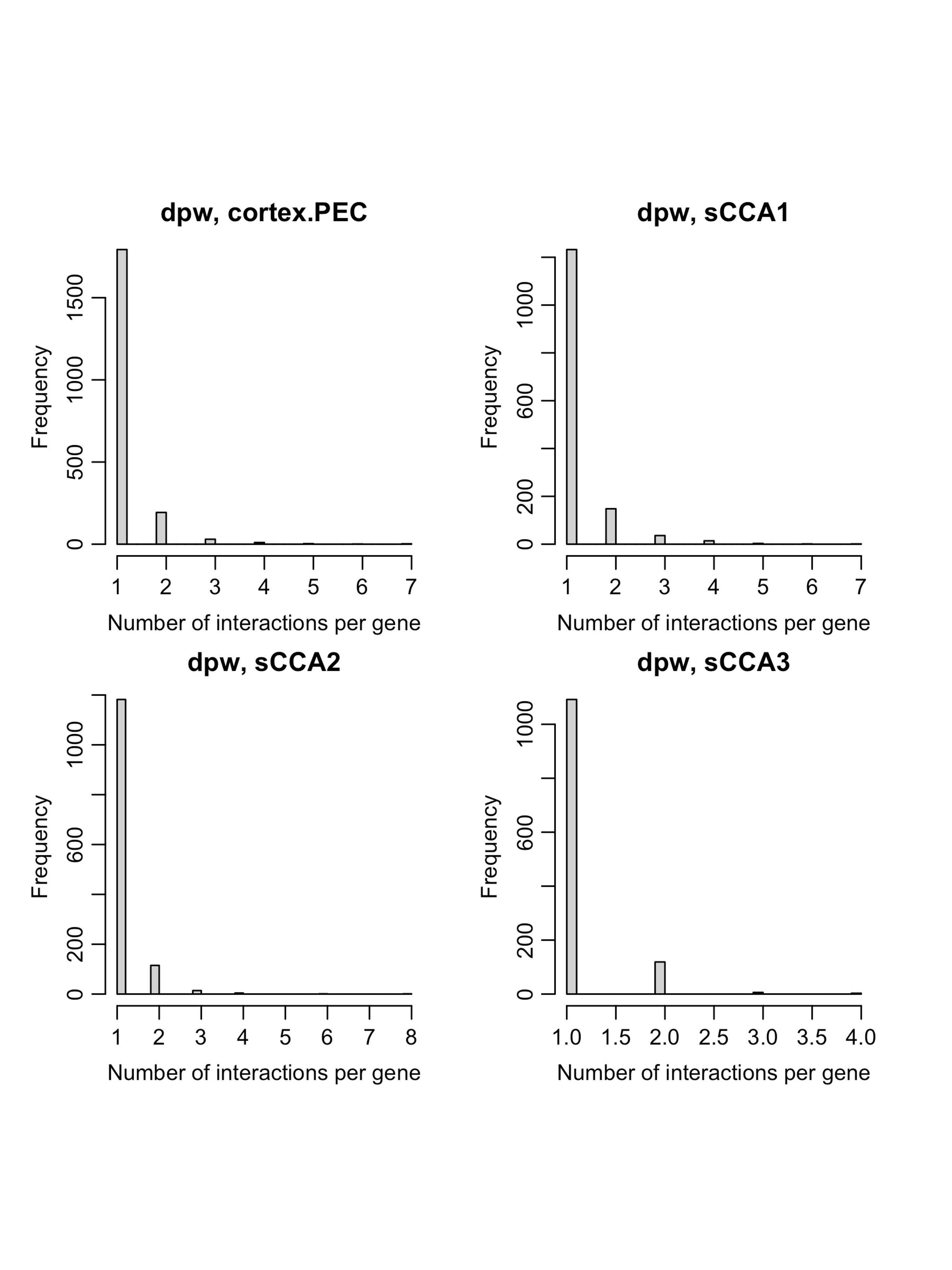

Supplement: S29 Fig — (TIFF) [file pgen.1010693.s030.tiff]

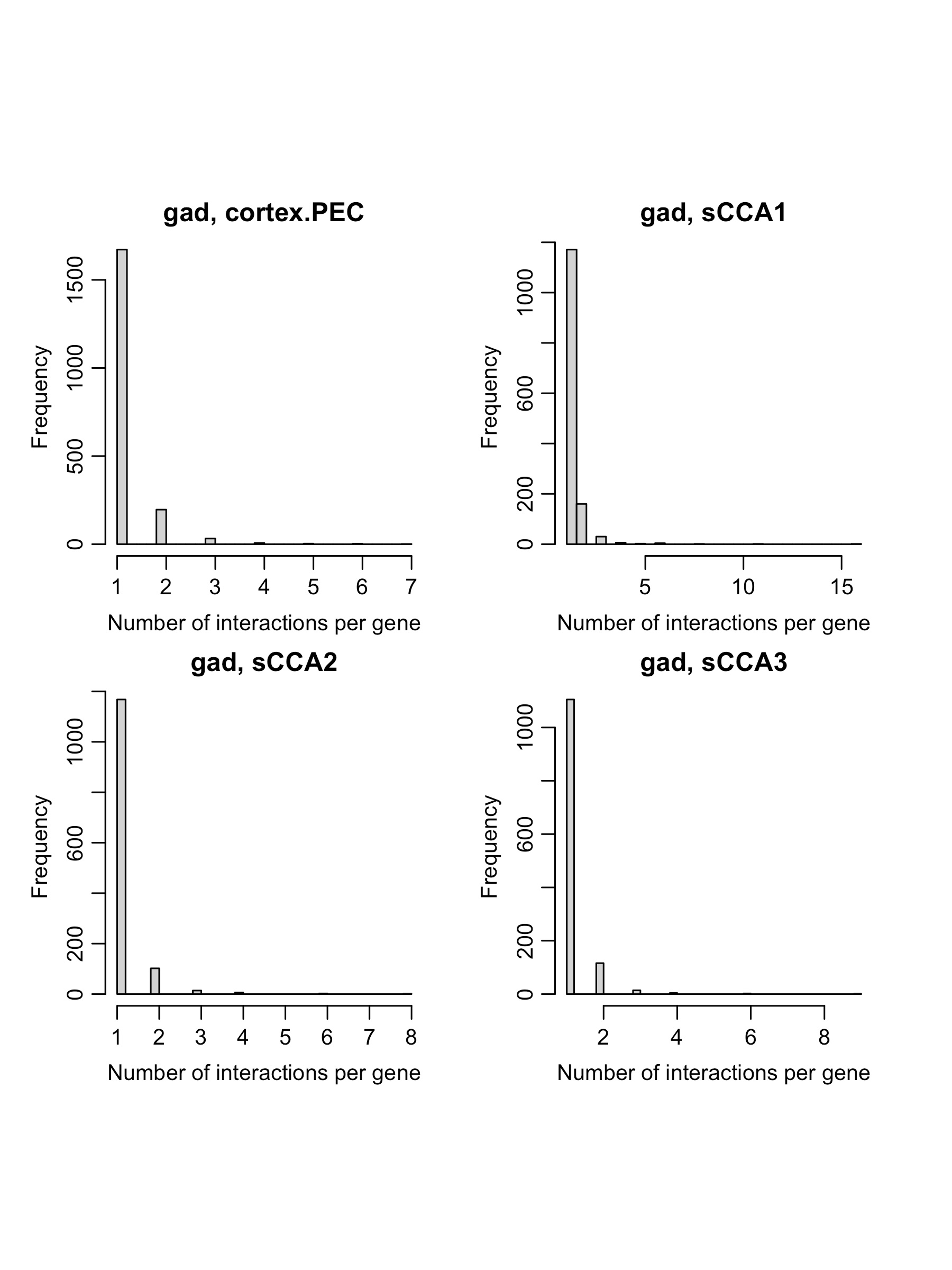

Supplement: S30 Fig — (TIFF) [file pgen.1010693.s031.tiff]

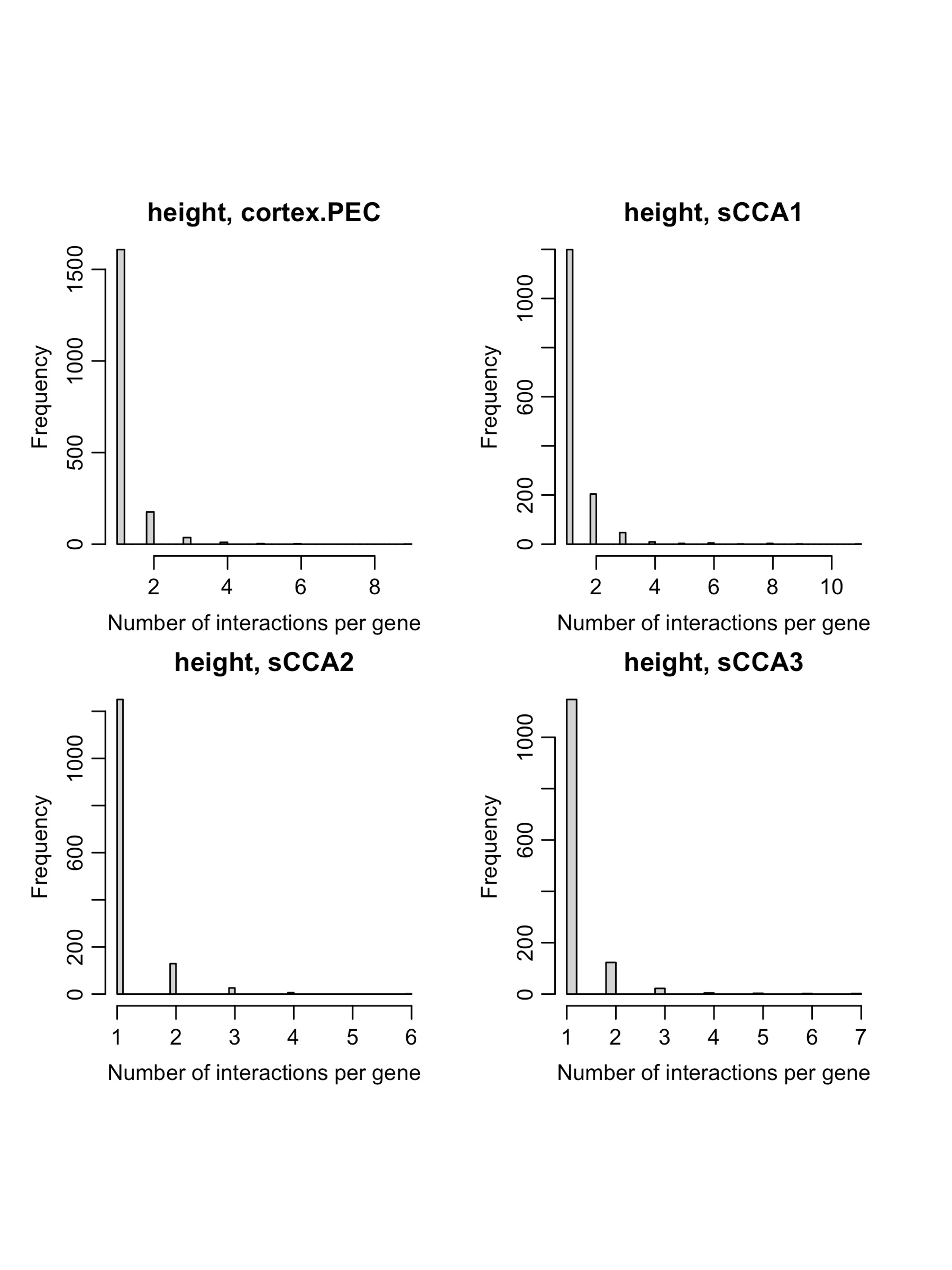

Supplement: S31 Fig — (TIFF) [file pgen.1010693.s032.tiff]

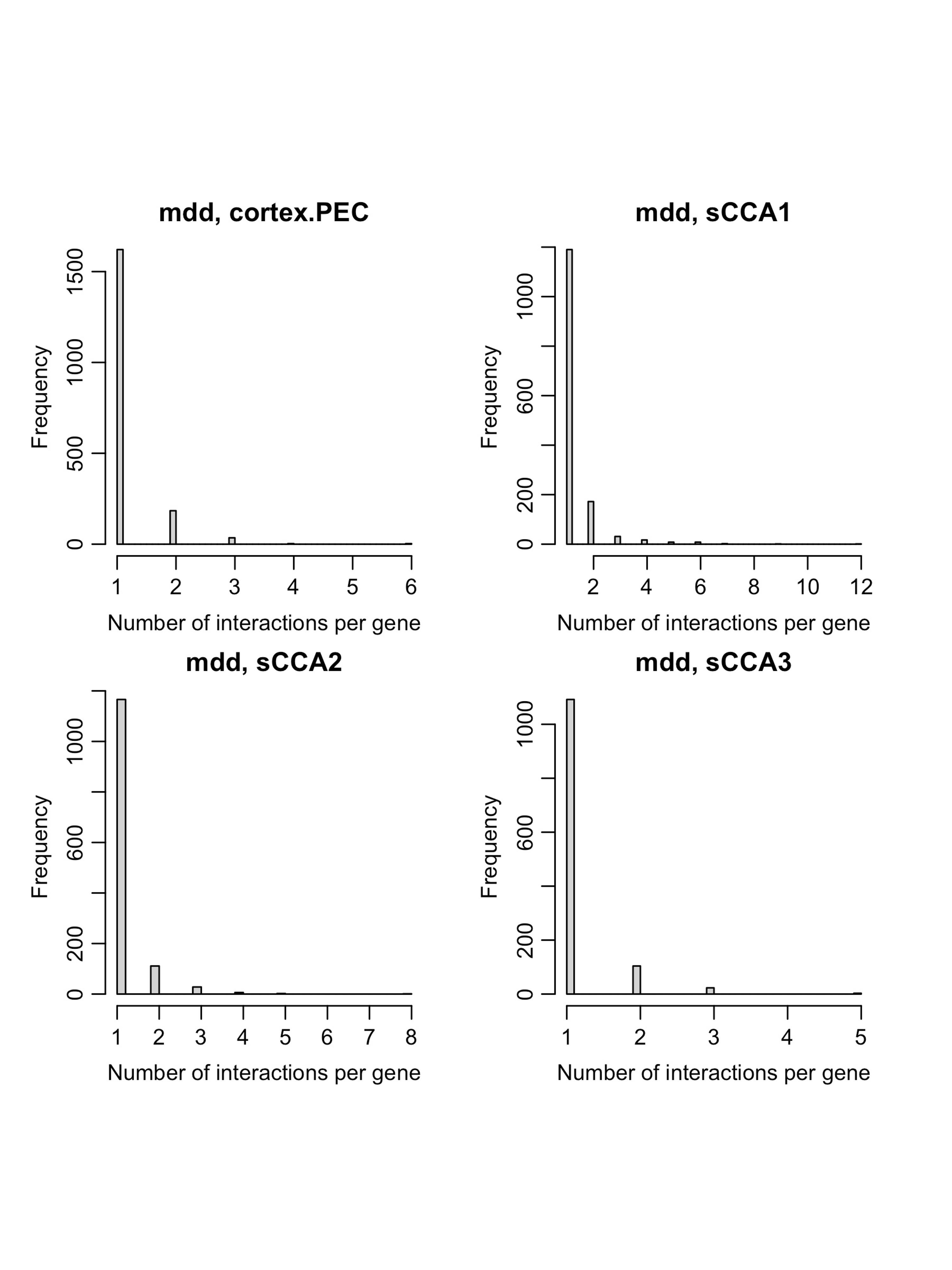

Supplement: S32 Fig — (TIFF) [file pgen.1010693.s033.tiff]

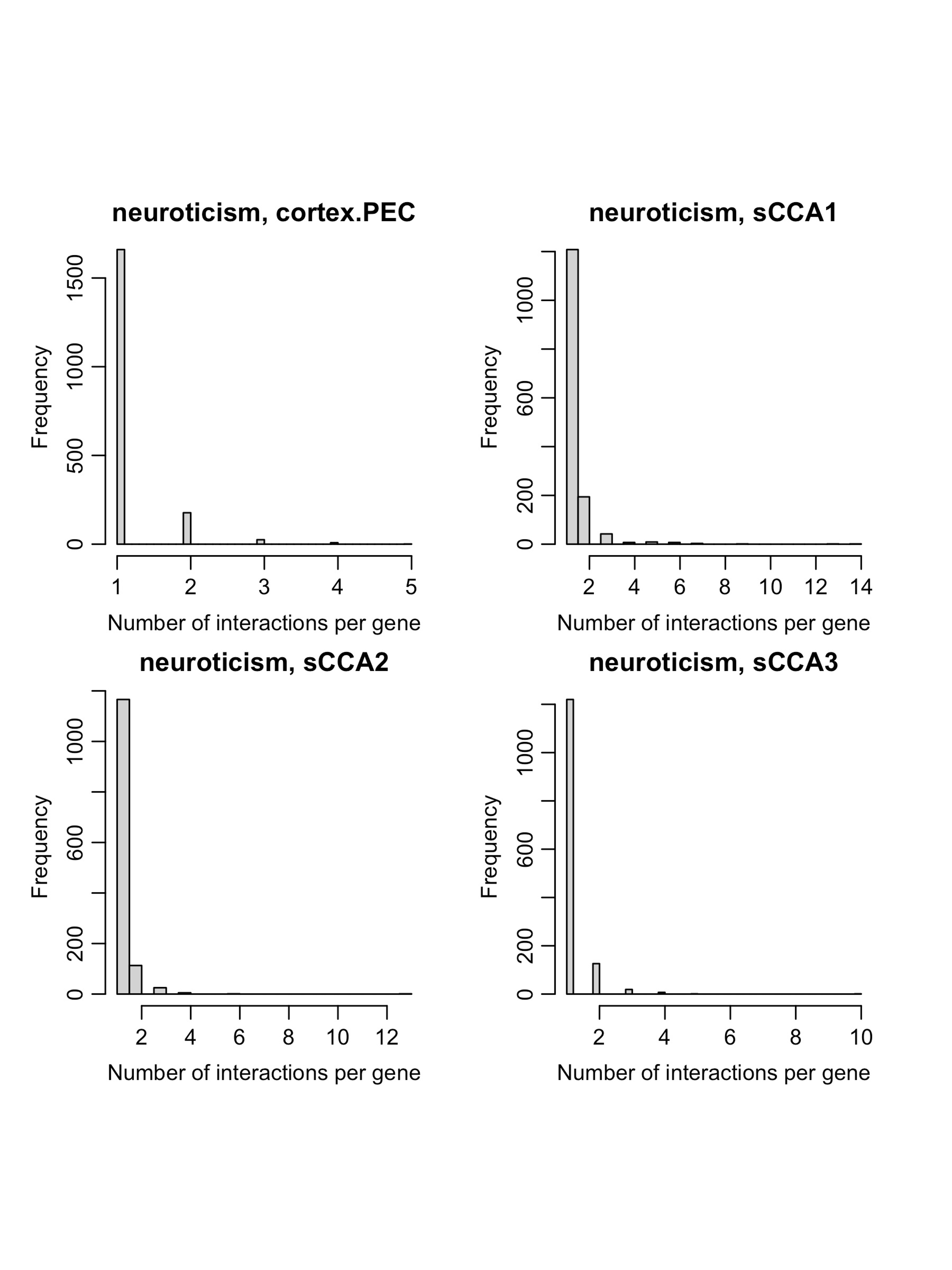

Supplement: S33 Fig — (TIFF) [file pgen.1010693.s034.tiff]

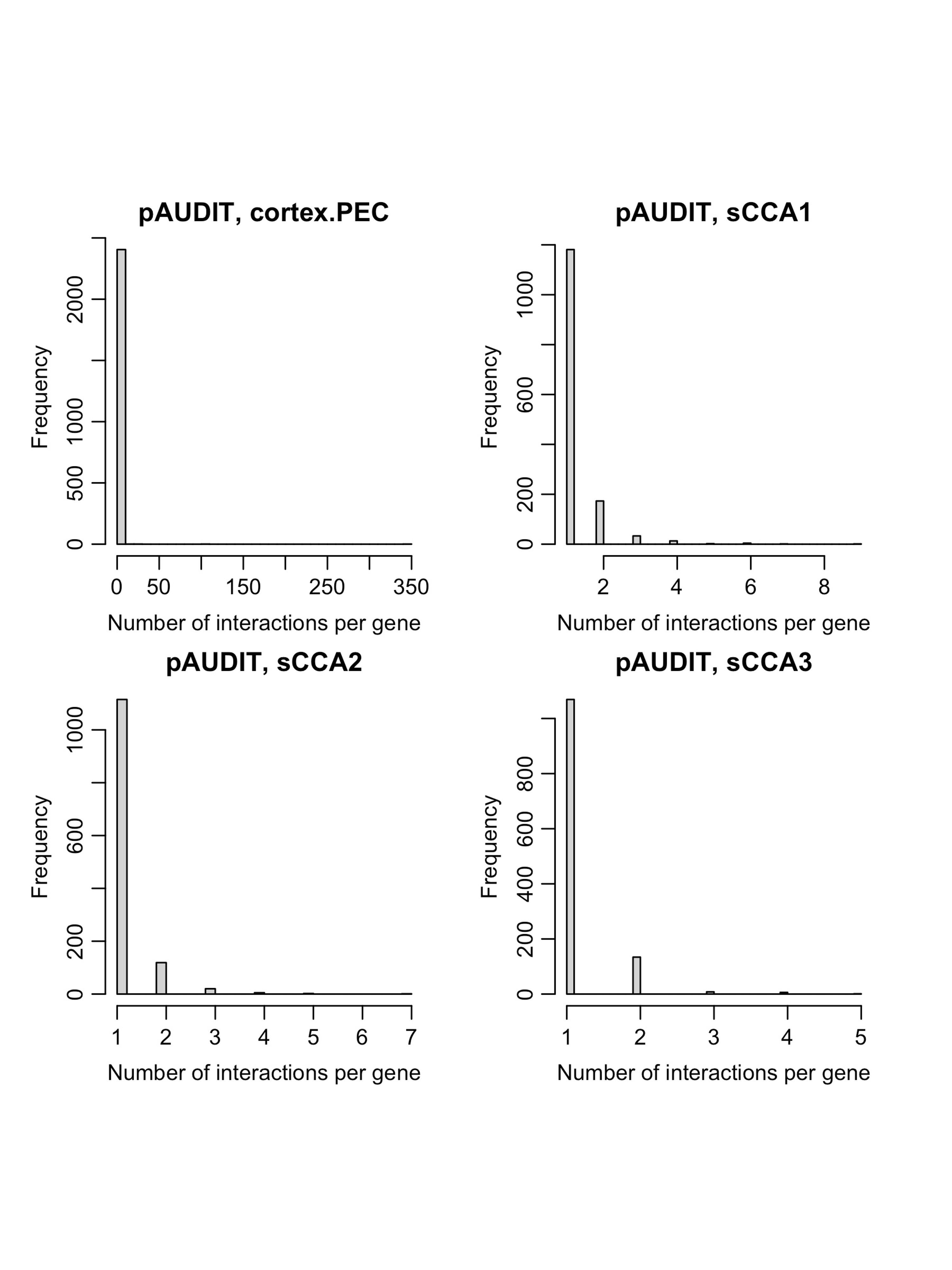

Supplement: S34 Fig — (TIFF) [file pgen.1010693.s035.tiff]

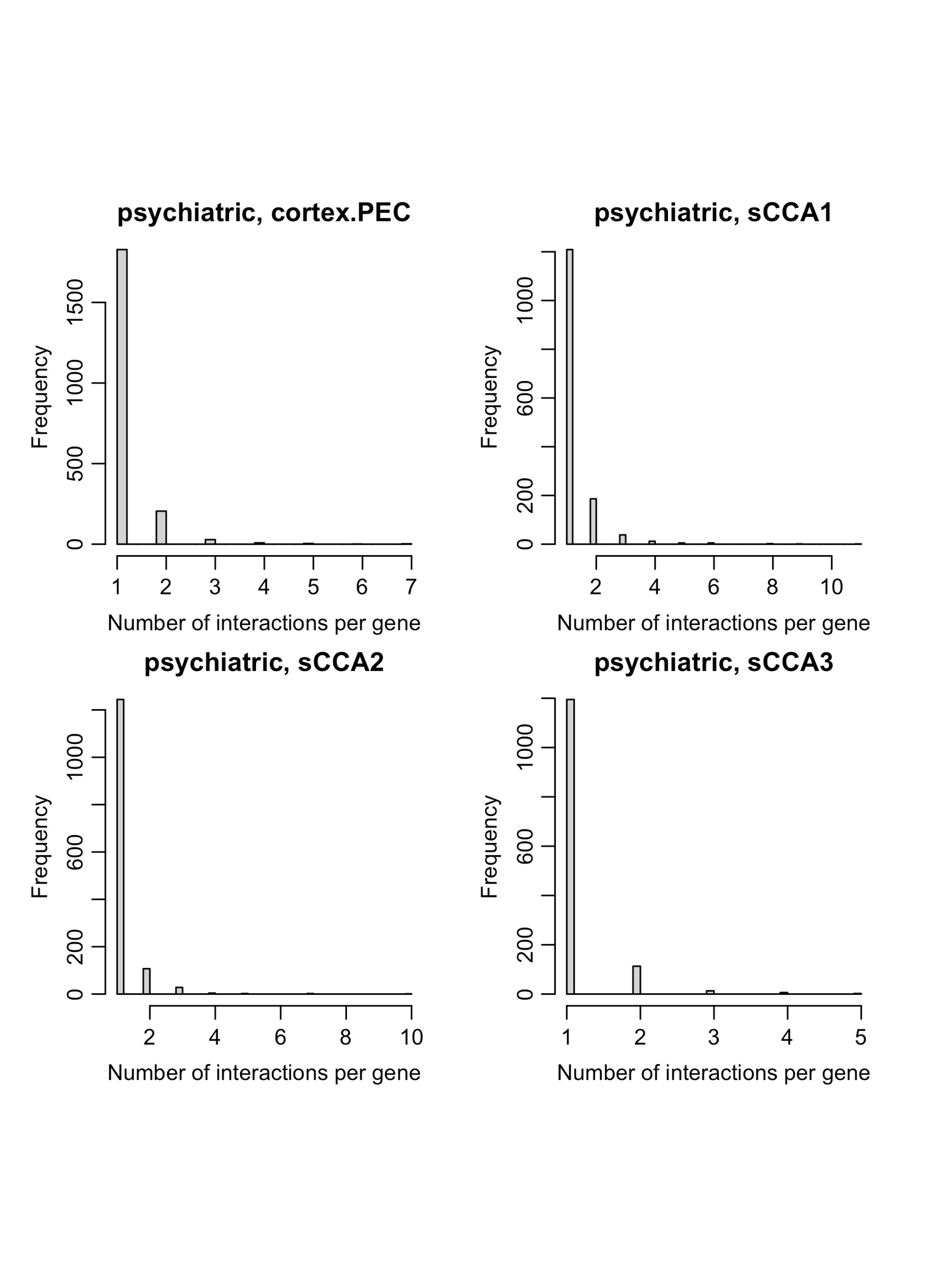

Supplement: S35 Fig — (TIFF) [file pgen.1010693.s036.tiff]

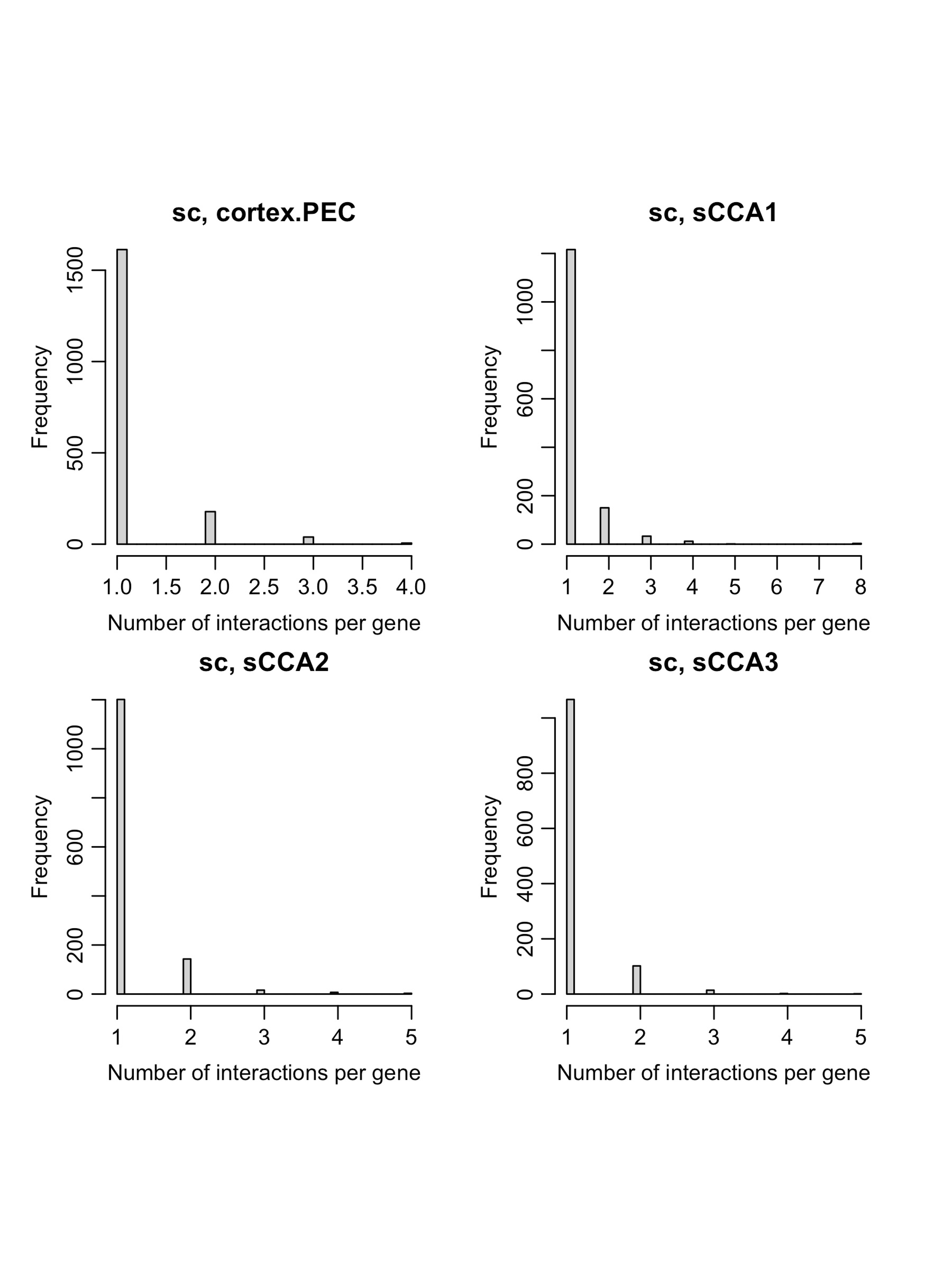

Supplement: S36 Fig — (TIFF) [file pgen.1010693.s037.tiff]

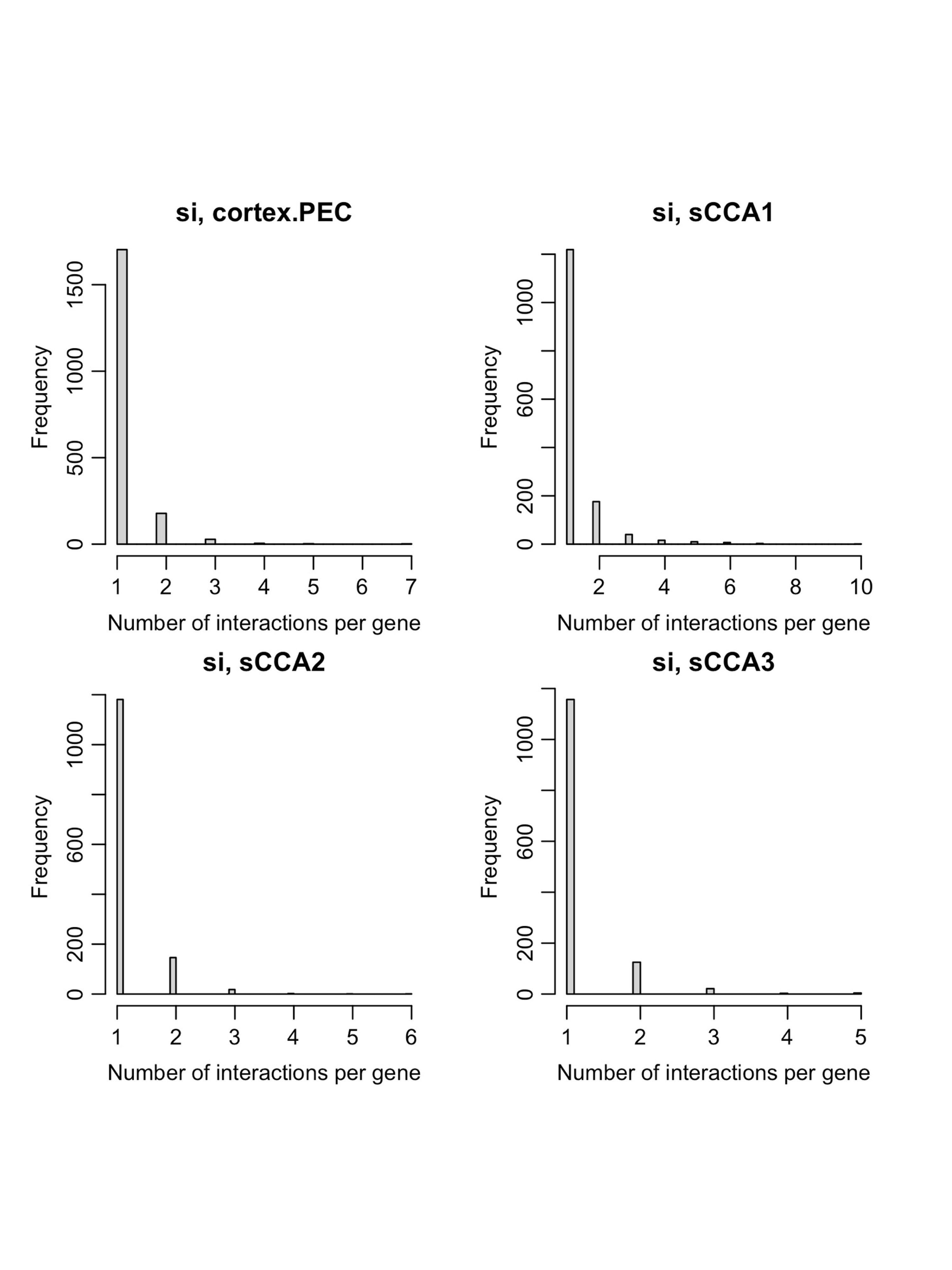

Supplement: S37 Fig — (TIFF) [file pgen.1010693.s038.tiff]

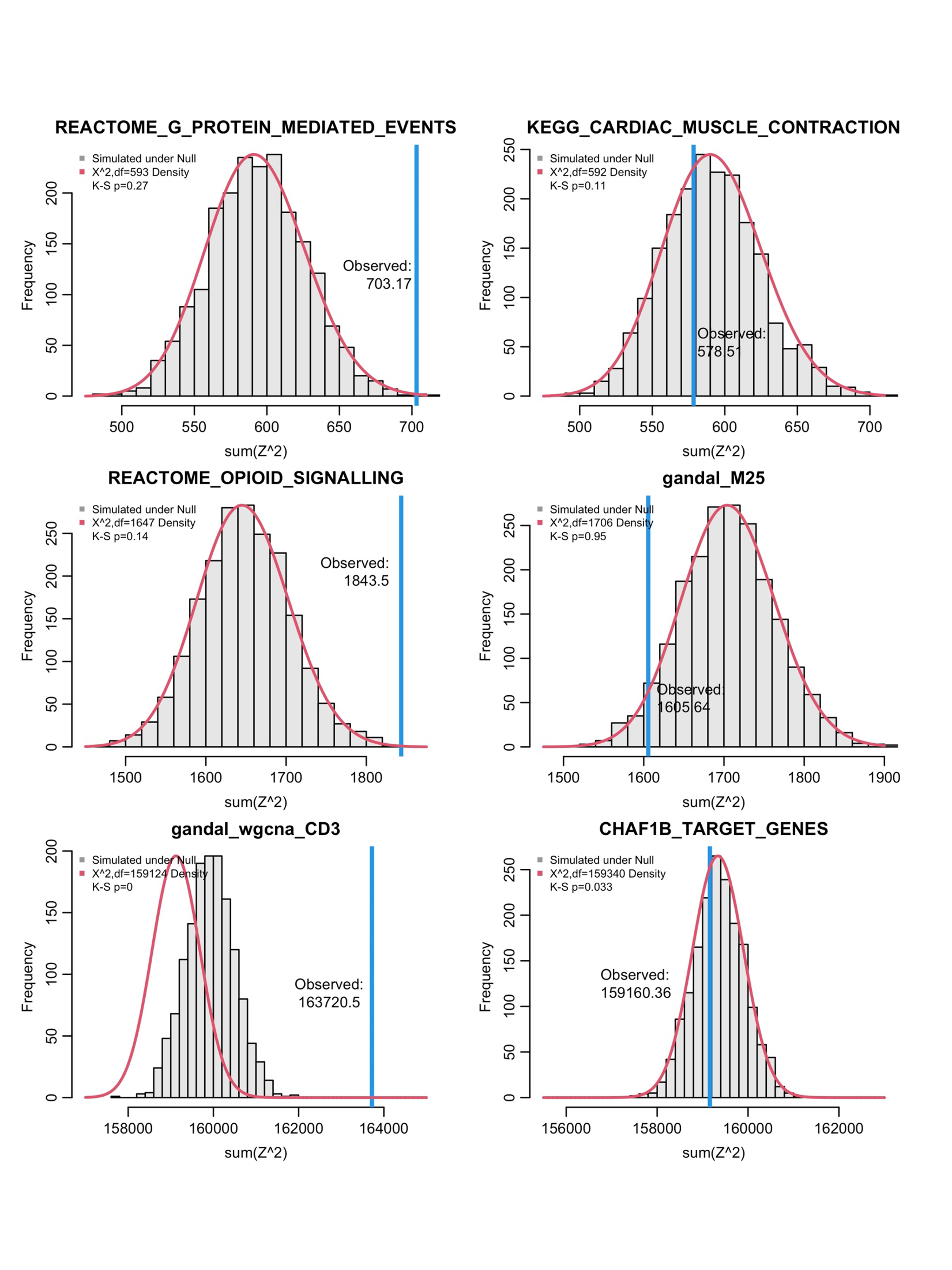

Supplement: S38 Fig — Observed value shown by blue line, while the red line represents the X2 density for the same df. These simulations show that for most gene sets, a standard X2 test is appropriate, but can be anti-conservative for large gene sets, likely when there is a true signal (e.g., gandal_wgcna_CD3 set). (TIFF) [file pgen.1010693.s039.tiff]

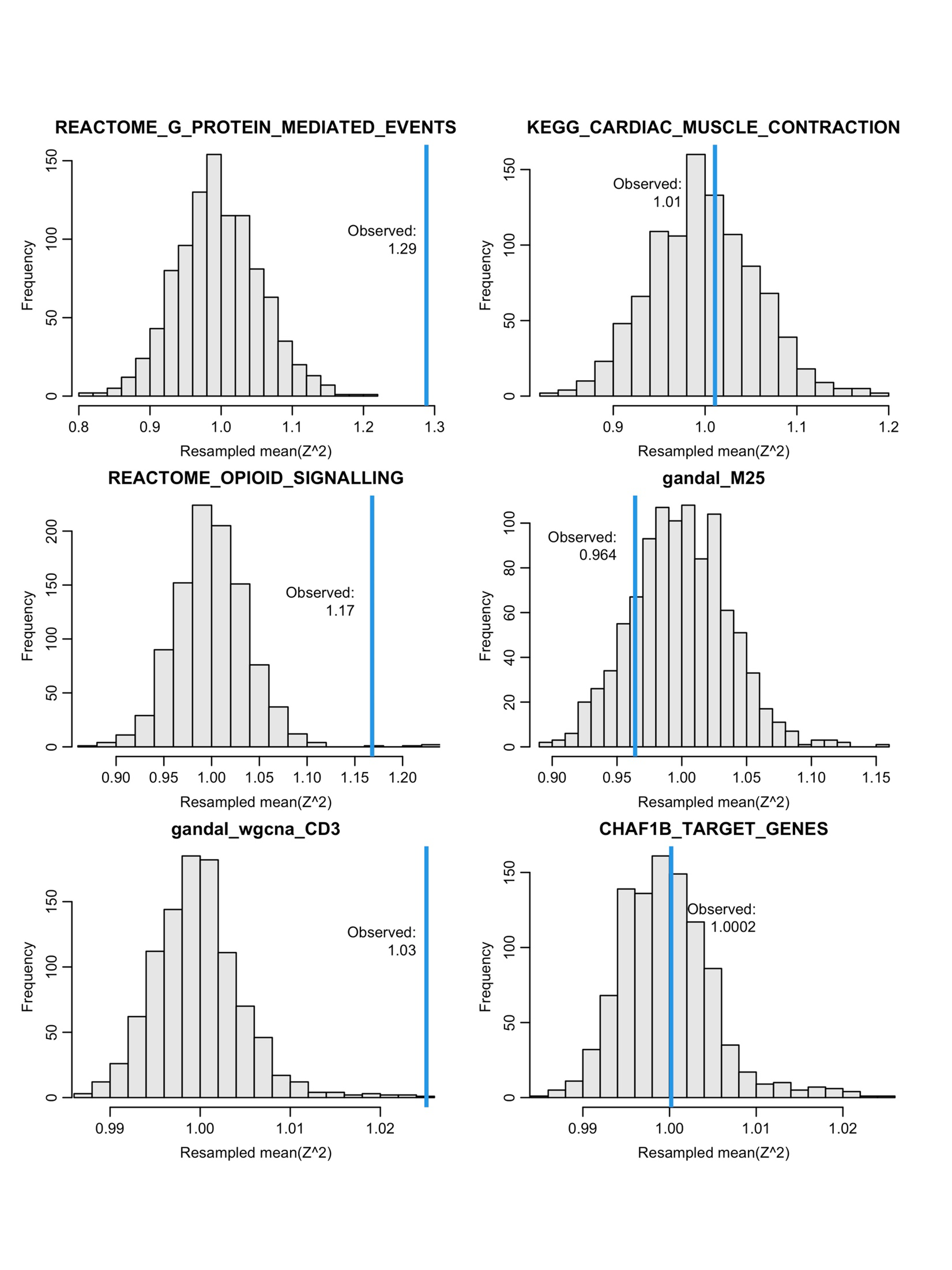

Supplement: S39 Fig — Observed value shown by blue line. These simulations show that for most gene sets, a random resampling approach recapitulates the results of a standard X2 test. (TIFF) [file pgen.1010693.s040.tiff]

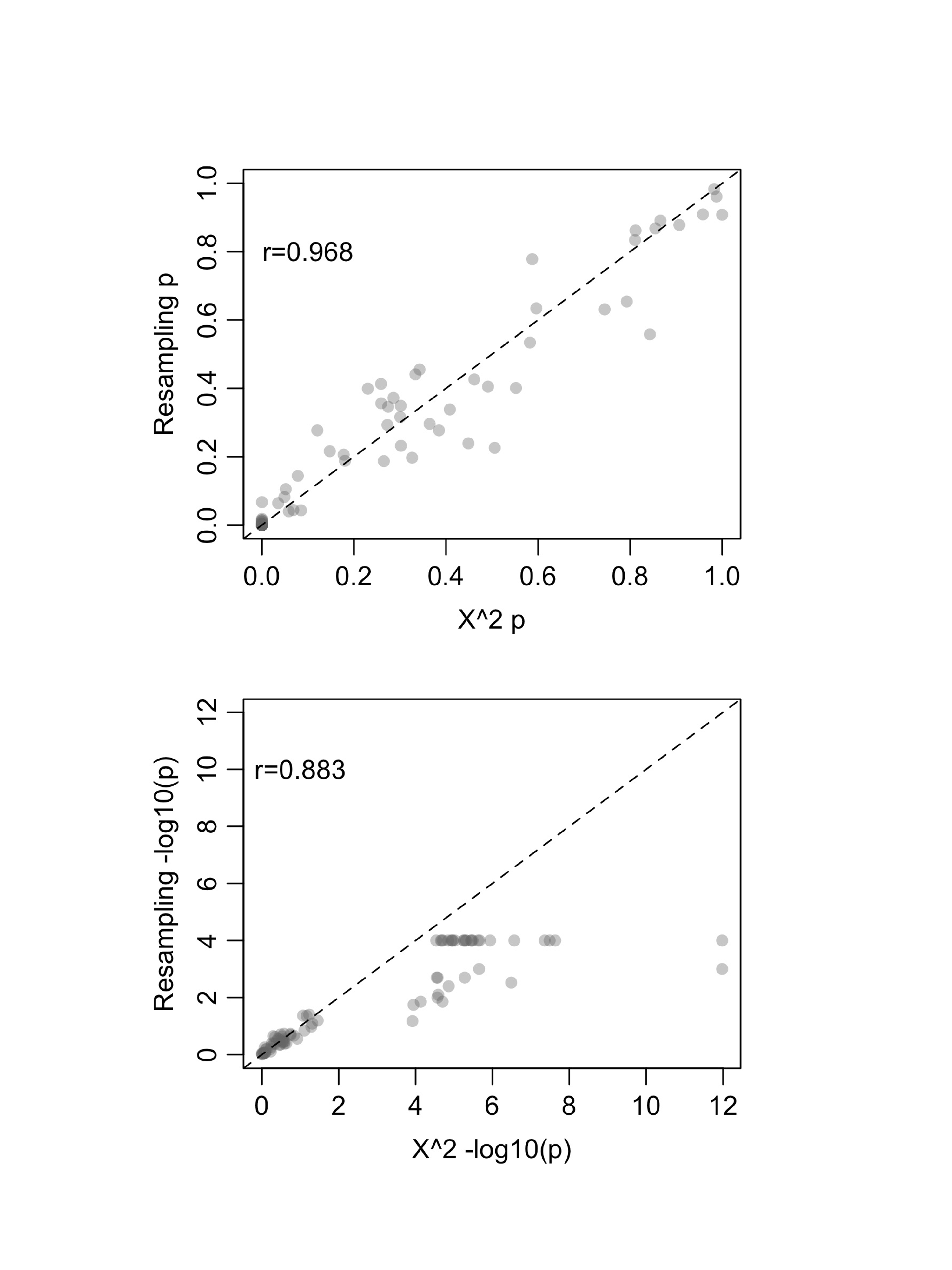

Supplement: S40 Fig — 1000 randomly resampled gene sets of the same size across a range of observed X2m p-values. Note that in the bottom panel, all cases where the resampled p-value was <1/1000 (i.e., none of the resampled sets had larger mean Z2 than the observed), -log10(p) was set to 4. (TIFF) [file pgen.1010693.s041.tiff]
