# Supplementary material for: Transcriptome-wide gene-gene interaction associations elucidate pathways and functional enrichment of complex traits
Source: PLoS Genet. 2023 May 22;19(5):e1010693. doi: 10.1371/journal.pgen.1010693 (PMC10237671; doi:10.1371/journal.pgen.1010693)
Supplement: Attachment — Submitted filename: Responses to Reviews.docx [file pgen.1010693.s052.docx]

Responses to Reviews

*We thank the editors and the reviewers for their comments and for the opportunity to revise and resubmit our manuscript. We have now fully addressed each comment, copied below. Responses to individual comments are in italics and we indicate the specific changes and rationale behind our changes.*

*One change we would first like to address is that I (LME) found a bug in my scripts that affected the way I treated smoking cessation in one cohort, which flipped the sign of the estimated effects. The bug was restricted to only that one trait and cohort; all others were not affected. I have now fixed that error and rerun those analyses for that phenotype in that cohort, as well as all downstream analyses (meta-analysis, enrichment) for smoking cessation. The manuscript has been updated with these new results, and Figs. 4-5, supplementary figures, and supplementary tables have all been updated. The change affected the genome-wide significant (when meta-analyzed) interaction association listed in the previous Table 1, which is now no longer significant (now p~4e-9), and the association has been removed from Table 1, as well as from highlighted descriptions in the text. I have also updated the meta-analyzed summary statistics deposited in Dryad. I apologize for the mistake, and note that it does not qualitatively change the findings or alter the big-picture implications of the manuscript.*

*All results are now fully, publicly available. All results and summary statistics are now available through dryad (*[*https://doi.org/10.5061/dryad.866t1g1tw*](https://doi.org/10.5061/dryad.866t1g1tw)*) or in the supplemental tables.*

*Thank you for the suggestion to use protocols.io. However, we had already begun preparing our analysis pipeline for GitHub and have made it publicly available (https://github.com/evanslm/TWIS). It is organized in a set of steps as a tutorial using publicly available 1000 Genomes data and FUSION-generated weights, as well as our meta-analyzed summary statistics (which are available through dryad). We hope this is acceptable, but would be able to move the tutorial scripts to protocols.io if you prefer.*

*We have used the PACE digital diagnostic tool for the images and have used the tool to ensure they meet PLoS requirements, and have uploaded the figures as tiff files separately.*

Reviewer 1:

In this manuscript, the authors develop a framework to measure epistasis by employing a flexible approach based on genetically-regulated expression and TWAS-based approaches. The method identifies and replicates some "hub" genes with multiple interactions. The method is ambitious and important and is a necessary step towards interrogating gene-gene interactions and their effects on complex traits. Overall, multiple questions still remain about the functionality of the method and the interpretation of the results.

*We thank the reviewer for their comments and suggestions below and we agree that assessing gene-gene interactions is an important aspect of complex traits.*

1. Line 144 - A natural question here is whether or not we expect epistatic genes to be correlated, even on the genetically-imputed scale. Can the authors comment on this and provide some intuition? It does make sense that correlation between the two variables (T1 and T2) or shared SNPs underlying the predictive model can lead to some level of collider bias, but do we not want to pick these genes up?

*Response: The reviewer notes an important limitation of our strategy to reduce the false positives due to LD. We agree that important epistatic effects could exist between nearby genes, and there are some good examples of this in the literature (we now cite one such example in line 161). Here, we view the drastic inflation of false positives as a major concern in our TWIS (see power analyses and supplemental figures S4-S6), and so we have prioritized limiting false positives at the expense of nearby loci or those with strong correlations.*

*We have now highlighted this and cited several examples of physically proximate loci known to interact, noting why we chose to limit our subsequent analyses, on lines 141-166. We report all results (including nearby and correlated pairs) in the publicly available summary statistics, should readers wish to explore those pairs in more depth.*

2. There are multiple different thresholds for correlations and P-values used throughout the paper. I would prefer the authors to aggregate these in a single figure/table and justify further.

*Response: As suggested, we have now included a table (Supplementary Table S3) that aggregates all the significance thresholds applied, the context in which we applied them, and further explanatory notes for each one regarding why we did or did not use them. Please see the new Supplementary Table S3 and lines 174-175 in the text.*

*For correlations of imputed gene expression, we have noted (line 170) that we chose a more conservative threshold than was suggested by the simulations (|r|<0.05 vs 0.1), which we feel is justified because we prioritize minimizing Type I error relative to Type II error.*

3. For replication of findings, only different GWAS were used. Since these cross-genome interactions may be affected by cell-type heterogeneity, replication across different weights may be important. Perhaps the authors could use the PsychENCODE weights and GTEx weights on the same GWAS to replicate?

*Response: As the reviewer notes, typically in a TWAS using different expression reference panels, a final omnibus test would be performed to assess overall support of expression-phenotype associations (Gusev 2016* [*https://doi.org/10.1038/ng.3506*](https://doi.org/10.1038/ng.3506)*). Our approach has made all gene-gene interactions tractable, but we still have a practical limitation of repeating all pairwise tests using an additional reference panel.*

*We recognize the benefit and added value of including multiple expression reference panels, but we also balance that with the added time that would be required to perform all tests across all traits with GTEx PFC reference panel data and then formally test using the omnibus test, as in standard TWAS. These analyses are intensive enough that we estimate it would take at least 3 dedicated months to complete the additional analyses.*

*We have, however, added to the “Limitations” section to state this important point, highlighting the possible heterogeneity in reference panels as the reviewer notes. Please see lines 418-423.*

4. Does the interaction term need to also be residualized by covariates? I suggest the authors run a sensitivity analysis for false positives/power by looking at residualizing the T1 x T2 term, as well, or provide some rationale otherwise.

*Response: As suggested here and also in response to comment R2.5 below, we have now performed a series of simulations in which we determine the false positive rates of the GxG term under a range of situations: covariates have effects on only traits, only expression, no effects on either, effects on both, under the null of no association between traits and GxG expression. Residualizing the T1xT2 separately can lead to strong rates of false positives when gene expression and covariates are correlated. However, residualizing the imputed gene expression terms (and then including the T1resid x T2resid interaction) never leads to inflated rates of false positives relative to the standard, full model. Please see the new Supplementary Table S2 and lines 138-139.*

5. Can the authors discuss an adaptive strategy to perhaps decrease the number of tests run?

*Response: Given the computational burden, this is a good suggestion, though we caution against using two-stage filtering approaches as described in the text. Reviewer #2 had a related comment, regarding the number of tests increasing due to the use of all tissues for all traits, despite some being biologically implausible or unlikely to be relevant, such as PFC expression for height, which we believe is a reasonable strategy. We now include a comment on lines 130-133.*

*Here, we include tests using all tissues in all traits for completeness, but a reasonable approach to reduce the overall number of tests would be to perform TWIS using only expression in biologically relevant tissues, cross-tissue expression measures, or in those tissues with, for example, significant S-LDSC h^2^_SNP_ enrichment for the trait of interest.*

6. Figure 2 is a little hard to understand. Do peaks in this Manhattan plot bear the same meaning as they do for GWAS/TWAS? For example, there's a peak on Chromosome 19 in Figure 2 (top). Do the authors consider this to be a hub gene?

*Response: The reviewer is correct in how they have interpreted the figure. We have now added additional text to the Figure 2 caption to clarify how we interpret these plots, which we hope makes it easier to interpret.*

7. A massive limitation of the paper is the lack of associated scripts. This is necessary for any level of replicability, especially when the scalability of the method is so important. Does the tool include ways to parallelize across other machines, or is this specific to the environments used by the authors?

*Response: We recognize this limitation and apologize for not having the GitHub repository ready by the time of initial submission. We have now made it public, and it includes scripts for each step, prepared as a tutorial using publicly available 1000 Genomes data and pre-computed TWAS weights that anyone can download. The core analysis scripts are in R, and we believe they can be applied to any compute environment with the required packages, but we also include example bash scripts to run them, with associated SLURM commands (the scheduler our compute environment uses, but which is also widely available on other computing clusters). The scripts can be run in a flexible way across different environments with variable numbers of compute nodes and CPUs available, though memory and time requirements will depend on the specific datasets individual users apply them to.*

*Within the GitHub repository are example files as well, and bash commands to download the publicly available 1000 Genomes and FUSION-computed TWAS SNP weights, using only a subset of the genome (chromosome 21) to make the tutorial tractable on any machine.*

*The enrichment analysis (E-TWIS) includes bash commands to download the now publicly available meta-analyzed summary statistics from our own results, then perform enrichment tests (χ^2^ and resampling) for several gene sets (including a couple of example gene sets of possible interest from ProteinAtlas as well as randomly generated sets from chromosome 21 to match the TWIS tutorial results).*

*The GitHub repository is* [*https://github.com/evanslm/TWIS*](https://github.com/evanslm/TWIS)

*All TWIS summary statistics (from the meta-analysis) are on Dryad at:* https://doi.org/10.5061/dryad.866t1g1tw

*We have also added these links on the website and data access sections of the manuscript. Lines 828-834.*

Reviewer #2: This is a well written and straightforward paper. The authors propose a new way to detect gene-gene interactions (in a broad sense). Instead of looking for SNPs in genes that interact with each other to affect traits, they imputed gene expression from eQTLs and looked for expression of pairs of genes that interact to affect traits. The model was simple, an interaction term (product of gene expression) was tested for significance in the presence of main effects. This model was fitted for every pairs (minus those with high correlation) thus termed TWIS (transcriptome wide interaction study). Several interactions were discovered and replicated in multiple datasets. Furthermore, the authors developed a method to test for gene set enrichment (E-TWIS) and found many pathways and networks enriched for TWIS signals. These led the authors to conclude that epistasis is likely widespread and the proposed methods may offer a useful way to explore gene-gene interactions.

Overall I find the paper easy to read, though it's a bit dense in details. The question is definitely an important one, i.e. what is the contribution of gene interactions to complex traits. However, the model as specified in the paper is very limited in scope and finds only one particular type of interaction for genes that are expressed in one tissue at a time. While it may seem like a good alternative to the computationally intractable search for SNP-SNP interactions, the two test for completely different hypotheses. These should be clearly pointed out and the limitations discussed. I detail below a few major points that need to be addressed/discussed:

*Response: We thank the reviewer for their positive assessment. We have edited the manuscript with attention to readability to address the dense nature of some of it, and we have also noted the reviewer’s point that SNP-SNP interactions and expression-based epistatic interactions may be testing different kinds of epistasis and different hypotheses. We detail these changes below.*

1) The SNP-SNP interaction tests look for DNA variants who interact to influence traits. The gene-gene expression interaction tests look for gene expression that interact to influence traits. A genuine SNP-SNP interaction is causal, a genuine gene-gene expression may not be causal and may reflect only secondary and reactive effects from the traits.

*Response: The reviewer makes an important point, and we agree that measured expression-based gene-gene tests could indeed reflect both causal or secondary/reactive effects. We believe this possibility is minimized by using the additive eQTL models in the TWAS framework, in which the genetically-predicted expression would not be influenced by environmental or secondary factors. We have noted this in line 411-422 in the “Limitations” section.*

2) The model tests for significance of the variable T1*T2. Because the imputed gene expression is based on an additive eQTL model, this means the proposed TWIS approach only discovers additive x additive interactions. There are a lot more types of interactions than additive x additive that would be missed by TWIS.

*Response: This is another good conceptual point, and we agree it is important to highlight the diversity of interactions that can exist. We have now also included this point in the “Limitations” section, noting that epistasis can reflect numerous other forms of interactions (e.g., dominance x additive, dominance x dominance, higher-order interactions). Please see lines 394-398.*

3) Line 215: It is not appropriate to test for a main effect when the interaction term is in the model. In the presence of a significant interaction, the main effect is meaningless because it's context dependent (depending on the other gene). The main effect is only relevant when there is no other term that includes it in the model.

*Response: We apologize for the confusion and our misstatement. The reviewer is correct that the main effect cannot be interpreted in the presence of an interaction, and we believe our wording emphasized the wrong aspect in this instance. Our goal was to point out that TWIS identified numerous gene-gene interactions that included novel genes, such as the GRIK1 exemplar we highlighted, that had not been identified in single locus GWAS or TWAS. We have removed the specific clause and reworded the sentence to be clearer. Please see lines 267-285 and also a related comment (R3.10) below and our response.*

4) I suggest the authors add plots to visualize the association between the top interactions and traits. For example, a 3-D plot with the x, y representing the two genes and the z representing the trait may be warranted here. Alternatively, plot phenotype against T1*T2.

*Response: As suggested, we have now made 3-D scatterplots for the 5 pairs of TWIS hits found in Table 1. These are new Supplementary Figures S21-S25, and use the (jittered for visualization) raw phenotype and imputed expression of the two genes.*

5）Figure 1. Both the traits and gene expression were adjusted for the same set of covariates. This may create spurious association. It's important to evaluate this in simulation. There are several scenarios: covariates have effects on only traits, only expression, no effects on either, effects on both. But there is not association between traits and expression, would you find false associations if both are adjusted by the same set of covariates.

*Response: This is an important concern, and as suggested here, and also in response to comment R1.4 above, we have now performed a series of simulations in which we determine the false positive rates of the GxG term under a range of situations: (i) covariates have effects on only traits, (ii) on only expression, (iii) with no effects on either, (iv) effects on both, and (v) under the null of no association between traits and GxG expression. Residualizing the T1xT2 separately can lead to strong rates of false positives when gene expression and covariates are correlated. However, residualizing the imputed gene expression terms (and then including the T1resid x T2resid interaction) never leads to inflated rates of false positives relative to the standard, full model. We believe these simulations address this concern. Please see the new Supplementary Table S2 and lines 138-139.*

*.*

6) Line 631: I'm not sure I agree that the sum of m Z scores in this context is a chi-squared with m df. The m Z scores are obviously not independent. This effect is more pronounced when the gene set is large and the authors propose a secondary resampling approach to guard against false positives. However, I think this should be the primary approach to be used for all gene sets, regardless of their size.

*Response: The reviewer is correct in that the sum of Z^2^ is not* $\chi^{2}$*distributed when the tests are non-independent, as they are here. With a small gene set, the effect is limited, and in simulation we found no evidence of test statistic inflation, but we found strong evidence of inflation in large gene sets (Figure S38).*

*We agree that the resampling approach provides the best way to assess gene set enrichment, but it is exceedingly slow when applied to genome-wide, exhaustive interaction tests. For exploratory analyses of thousands of gene sets, as we performed in our E-TWIS analyses, it is too slow to realistically use. However, we see this as an opportunity to first identify any sets with possible evidence of enrichment using the fast* $\chi^{2}$*approach, then follow up these with more rigorous resampling tests. We have clarified in the text on line 303 and 386 to recommend applying the resampling approach to any significant set or those of specific interest, rather than just large, significant sets. Our code on GitHub, however, by default now performs both tests automatically. We hope this is an acceptable compromise between efficiency in performing exploratory tests and rigorous testing of gene sets of particular interest.*

Reviewer #3: In this manuscript, Evans and coauthors introduce a new approach named TWIS to find gene-gene interactions affecting complex traits. This extends the TWAS approach by testing pairwise interaction effects between imputed gene expression on complex traits of interest. The main advantage of the proposed approach compared to existing methods is the reduced multiple testing burden (due to the test being at the gene-level rather than the variant-level) without making any pre-filtering based on, for example, the significance of a single gene analysis. The authors apply their proposed approach to 12 complex traits using large datasets and found a few significant interactions. The authors also developed a procedure to test for enrichment of gene-gene interactions in predefined sets. The results are somewhat underwhelming, given that only 1 interaction replicated in an independent dataset and only 6 interactions were significant at the meta-analysis across datasets. However, it is refreshing to see researchers focus on gene-gene interactions in humans, which have been documented extensively in model organisms, rather than neglecting them a priori. Furthermore, the authors performed extensive simulations to show that their approach controls for false positives, but has low power (as expected for interaction effects) at current sample size. I believe this is a promising approach and is of interest to the broad human genetics community, however, I do have a few comments.

*Response: We thank the reviewer for their positive assessment and their useful suggestions below. We acknowledge the power limitations, and have endeavored to pull together as many cohorts as is feasible. Despite this limitation, we agree with the reviewer that extending these studies to human systems and understanding interactions can still provide key biological insight and are worth exploring.*

1 – Line 65-66. I think it is important to distinguish between gene action and contribution to variance components. See Huang and Mackay 2016 PLOS Genetics

*Response: This is an excellent point, and we agree it is important to distinguish these concepts. We have now added a sentence to this effect on lines 67-70, as suggested.*

2 – Line 108. What was the rational for choosing these particular traits?

*Response: These traits were chosen because they were available in large, public datasets, and because they span a range of trait domains, from biometric to psychiatric, and thus we feel would be a reasonable set to demonstrate the utility of the approach. We have added a sentence to this effect at the beginning (page 2) of the supplementary information.*

3 – Line 115-119. I am not sure about the importance of PFC for height and BMI, for example. So, the results for those traits may be affected negatively by the choice of these tissues. Maybe an enrichment-type analysis (e.g., S-LDSC) could be performed to choose the most relevant tissues?

*Response: This is a valid point, and we thank the reviewer for suggesting a reasonable approach to identify relevant tissues, S-LDSC. We have now included a statement to this effect on lines 130-133. Please also see our response to the related comment R1.5 above.*

4 – Line 134. Is it really N(0,1) or is it N(0, sigma^2)?

*Response: Yes, the reviewer is correct and we apologize for the typo. It should be sigma^2, and we have fixed the mistake (line 149).*

5 – Line 142. I am assuming you mean LD rather than linkage? I would also point out that LD is between the variants constituting the expression prediction model. However, it is important to note that LD is only one possible source of imputed expression correlation. From Weinberg et al 2019 Nat Gen “A gene pair can have correlated predicted expression if the same causal eQTL regulates both genes or if two causal eQTLs in LD each regulate one of the genes”. In general, I find that LD and linkage – two very distinct concepts – are sometimes used interchangeably throughout the manuscript adding to the confusion. Please make sure that the correct term is used in the appropriate context.

*Response: We apologize for the imprecise use of LD and linkage. We have corrected this throughout the manuscript. We have also noted that shared, causal eQTLs can lead to correlated predicted expression. Please see line 158 and the “Limitations” section lines 411-417.*

6 – Line 146-148. Are there cases where |r| < 0.05 within 1 Mb? And if so, could such cases lead to false positives? My understanding is that physical distance per se doesn’t inflate false positive rate — it is the high correlation that does inflate, which may be due to physical distance. So, the authors could remove the filter based on distance?

*Response: The reviewer is correct – all of our simulations and also others’ published work has pointed to high correlation of binomially distributed predictors in the context of additional error that leads to false positive inflation, not physical proximity per se, and our distance and correlation thresholds may be removing biologically important instances of epistasis. Comment R1.1 above notes this could be the case when the correlation |r| is > 0.05, for example. Among our simulations, we found situations as in Supplementary Figure 6 (bottom), in which there may be LD between causal SNPs and those used in expression imputation may lead to positive TWIS associations, even if the imputed expression correlation is |r|<0.05. Given the drastic inflation of false positive TWIS associations when physically nearby genes are tested (Figure S4), we have therefore prioritized reducing the false positive rate, but it does come with limitations, as noted in our response above, and we acknowledge that these depend on somewhat subjective risk tolerance criteria. We have also released all TWIS summary statistics including pairs that were filtered from subsequent analyses, if readers wish to explore those pairs further.*

7 – Line 154-163. I find this part pretty confusing. For example, there are 4 significant interactions for pAUDIT in Table 1 but only 3 interactions above the dashed line in Fig 2. Also, in Fig. 2 either the authors present all the significant results (i.e., including SC too) or they pick only one trait as an example. Also, on line 162 the authors say “Of the five significant in the final meta-analysis…”, but they are 6, if I understand correctly. Please make the whole paragraph clearer.

*Response: We apologize for the confusing structure and wording, and for a typo in the text. We have reworded the text in this paragraph to be clearer in describing the pairs that were significant in any stage (discovery, replication, final meta-analysis), and added a supplemental table (S4) that contains all 17 pairwise interaction summary statistics. We have also broken this paragraph into two to distinguish between the overall results across all tissues and traits, and the 4 interactions that were significantly associated with pAUDIT, again clarifying the initial wording. We hope these have made this section more understandable.*

*Please see lines 177-187 and Table S4.*

8 – Line 186. I am not sure what you mean by “imperfect” expression imputation. If it is r^2 < 1, that will never happen for genes with h^2_g < 1. Remember that r^2 <= h^2_g since only SNPs are used to predict expression. So, you could have r^2=h^2_g<1 which would be perfect imputation. So, I would define imperfect imputation as r^2

*Response: We have now clarified that imperfect imputation here reflects r^2^<h^2^<1: “…in the context of imperfect expression imputation (where imputation r^2^<expression heritability, the maximum accuracy of the genetic prediction) across …” Please see line 155-156.*

9 – Line 190. Fig. 3 says p<1e-6. Which is the correct one?

*Response: We apologize for the confusing sentence. The distributions of number of suggestive interactions applied the p≤1e-5 cutoff (Figures S26-S37 & Table S5). Figure 3 applied a more stringent p≤1e-6 cutoff, solely for visualization purposes. The new Table S3 outlines the thresholds applied for each analysis, including notes on the rationale behind the specific threshold. We hope the reworded text and the new Table S3 clarify the issue.*

10 – Line 211-227. If I understand correctly, these are two different situations. The first (GRIK1 - CPD association) is an example of a main effect not being identified without an interaction in the model (probably because the interaction explained some variance that went into the error in the single gene model). The second (% of genes recovered by the single gene model) is an example of genes that have a significant interaction association without their main effects being significant. If my understanding is correct, this is comparing apples to oranges. A better comparison would be checking the % of genes with significant MAIN effect at TWIS that can be recovered by TWAS. If my understanding is incorrect, please clarify.

*Response: We apologize for the confusion and our misstatement. The reviewer is correct that these were comparing two different scenarios, and our wording emphasized the wrong aspect of the comparison. Our goal was to point out that numerous genes were identified in TWIS, specifically through their significant interaction terms, that have never been identified in single locus GWAS or TWAS, and we were trying to highlight* GRIK1 *as an example. We have removed the specific clause, and reworded the sentence to be clearer. As noted by R2.3 above, comparison of the estimated main effects in an interaction model and the estimate in a model without interactions would compare different parameters, and we have removed the wording that suggested this. As the reviewer notes, the interaction likely explains some variance that goes into the error term in the single gene model, and so we believe it is valid to estimate the proportion of genes identified with significant interactions that would have also been identified in single locus models as a way to highlight the novel loci that influence a trait which can be identified when incorporating interactions, and therefore uncover new aspects of the trait’s biology that would have been missed in standard TWAS or GWAS. We hope the changes in the text (lines 267-285) have clarified the section.*

11 – Line 239-242. Why did you choose these specific gene sets?

*Response: Our goal here was to perform an exploratory assessment of interaction enrichment and we identified sets to span a range of functions and possible biology. We have now added the following at line 308: These represent a wide range of types of gene sets, across a wide variety of functional pathways, tissue expression specificity, and possible interactions (e.g., WGCNA modules), for an exploratory analysis of interaction enrichment.*

12 – Line 276. Not sure you can conclude that from those two observations, especially given that the actual significant interactions are only 6 and for 3 of the 12 traits analyzed.

*Response: We have now changed the wording to tamper the certainty in the statement and acknowledge our speculation, from “These two findings imply that epistasis is frequent, with key hub genes yet to be identified.” To “These two findings suggest that epistasis may be frequent, and hub genes may yet be identified.” Please see line 356 and line 36.*

13 – Line 422. From the fastLm manual “However, Armadillo will either fail or, worse,
produce completely incorrect answers on rank-deficient model matrices whereas the functions from the stats package will handle them properly due to the modified Linpack code”. This might not have been a problem in your analysis, but is something to keep in mind when testing for interactions.

*Response: We thank the reviewer for bringing this to our attention. We, fortunately, believe this is not an issue given our strategy of first, residualizing the predictors and the phenotype on the (full-rank) covariate matrix, and second, removing pairs in which expression is correlated. These two steps should ensure that the model matrix in the actual TWIS analysis (T1resid+T2resid +T1resid *T2resid) is full-rank. The issue of strong LD between genes would be one instance where the model matrix would be rank-deficient, but we remove those pairs from analyses due to the high false positive rate.*

14 – Line 504-505. I think you mean decreasing interaction PVE also decreases the power. And adding prediction error further decreases power. Is that right?

*Response: Thank you for pointing this out. Both factors decrease power, and we have now changed line 628 to clarify this point.*

15 – Line 523. Genes are not in LD — variants are. Again, please make sure the appropriate terms are used.

*Response: We apologize for the imprecise use of LD and linkage. We have corrected this throughout the manuscript.*

16 – Could the authors try to find the variant-variant interaction(s) underlying the significant gene-gene interactions? For example, by testing all the possible pairwise interactions between the variants making up the prediction models for the two genes. The significance threshold would be reduced like in a candidate gene approach. Power might still be an issue but it is worth trying.

*Response: Thank you for this suggestion, and as suggested, we have now run all additive-by-additive SNPxSNP interaction associations for the SNPs surrounding (+/- 500Kb) the five pairs of genes listed in Table 1, using a similar model. The final meta-analyzed results are also now publicly available in the dryad repository. While several pairs were suggestive (p<5-8), none reached the genome-wide significance threshold we identified of 5.86e-10, but we note that with sufficient sample size and power, TWIS is a reasonable approach to identify a restricted set of genes around which all SNPxSNP interactions can be tested. Please see lines 815-823 of the Methods and 188-206 of the Results.*
