# Supplementary material for: Transcriptome-wide gene-gene interaction associations elucidate pathways and functional enrichment of complex traits
Source: PLoS Genet. 2023 May 22;19(5):e1010693. doi: 10.1371/journal.pgen.1010693 (PMC10237671; doi:10.1371/journal.pgen.1010693)
Supplement: Attachment — Submitted filename: Responses to Reviews.docx [file pgen.1010693.s053.docx]

Responses to Reviews

*We thank the editors and the reviewers for their comments and for the opportunity to revise and resubmit our manuscript. We have now fully addressed each comment, copied below. Responses to individual comments are in italics and we indicate the specific changes and rationale behind our changes.*

Reviewer #2: The authors have adequately addressed most of my questions. However, when I asked (#4) for visualization of the significant interactions, I suggested 3D plot or 2D plot (phenotype versus T1*T2). I expected the authors to at least make some effort to make sense of the plot. They did make a 3D plot but did not try to explain any of it other than saying there is a figure. I believe this is a lost opportunity to make the paper more accessible. I suggest the author in their text explain how the figure is showing association between the phenotype and T1*T2. It does not have to be a 3-D plot, which apparently isn't very intuitive. Perhaps phenotype ~ T1*T2? or any other ways you find useful to visualize the association.

*We have now updated Figures S21-S25, as suggested by a reviewer, to provide a better visualization of the interaction of imputed expression effects on the traits. We hope this provides a more intuitive way to visualize and interpret the results. We have also added interpretation of these effects in the text, as requested by the reviewer, describing the directions of effects of one gene conditioned on the high vs. low expression of the second gene. Please see updated Fig. S21-S25 and lines 194-195, 200-202, and 207-208.*

Reviewer #3: I thank the authors for addressing my comments -- I am generally satisfied with the revision. One minor issue though. There are still a few places in the manuscript where the authors say "genes in LD", for example lines 584, 610, 611, 622, 663, 668. Variants are in LD, not genes. Please correct.

*We have now updated the text to specify that the SNPs are in LD. Specifically, we have changed L589 (to “genes when local SNPs are in LD.”), L597 ( to “correlated due to LD of SNPs…”), L615 (to “When SNPs affecting gene expression are not in linkage disequilibrium…”), L616 (to “…if the SNPs affecting expression are in LD…”), L628 (to “Gene pairs physically or with unlinked SNPs affecting them are…”), L673 (to “as a proxy for pairs in which SNPs are possibly in LD vs. those not in LD.”), and L678 (to “…inflation due to LD between SNPs nearby the genes…”).*
